# Sunlight-assisted activation of peroxymonosulfate using NiFe_2_O_4_ supported on a 2D g-C_3_N_4_ nanosheet heterojunction for the degradation of methylene blue in wastewater

**DOI:** 10.1039/d6ra06402g

**Published:** 2026-10-05

**Authors:** Siphumelele Thandokwazi Mkhondwane, Yogendra Kumar, Vinod, Viswanadha Srirama Rajasekhar Pullabhotla

**Affiliations:** a Department of Chemistry, University of Zululand Private Bag X1001 Kwa-Dlangezwa 3886 South Africa siphumelele.mkhondwane@ru.ac.za; b Department of Chemistry, GLA University Mathura Uttar Pradesh 281406 India; c Department of Chemistry, University of Lucknow Lucknow-226007 Uttar Pradesh India

## Abstract

This study explores the utilization of a 0D/2D NiFe_2_O_4_/g-C_3_N_4_ heterojunction for the photocatalytic activation of peroxymonosulfate (PMS) to degrade methylene blue in wastewater. The NiFe_2_O_4_/g-C_3_N_4_ composite was synthesized from 0D NiFe_2_O_4_ and 2D g-C_3_N_4_ using wet impregnation and calcination. The NiFe_2_O_4_/g-C_3_N_4_ composite was then characterised using various spectroscopic and microscopic techniques and used for the photocatalytic degradation of methylene blue in the presence of PMS (1 mL, 0.1 M). The NiFe_2_O_4_/g-C_3_N_4_ composite showed a higher degradation efficiency of 97% compared to NiFe_2_O_4_ and g-C_3_N_4_ alone, which showed 69% and 53%, respectively, after 90 min of degradation. The concentration of PMS was optimized across 0.2 M, 0.4 M and 1.6 M, yielding degradation efficiencies of 99%, 91% and 74%, respectively; this indicates that the highest degradation performance is obtained at 0.2 M. Subsequently, the pH was also optimized, with pH 9 and 11 achieving 100% degradation efficiency. The radical quenching experiments showed that sulfate radicals (SO_4_˙^−^) were the dominant reactive oxygen species (ROS) responsible for the degradation of methylene blue. The study showed that NiFe_2_O_4_/g-C_3_N_4_ has the potential to be used as a catalyst for PMS activation to degrade organic pollutants in wastewater under light irradiation.

## Abstract 2

This study explores the utilization of a 0D/2D NiFe_2_O_4_/g-C_3_N_4_ heterojunction for the photocatalytic activation of peroxymonosulfate (PMS) to degrade methylene blue in wastewater.

## Graphical abstract

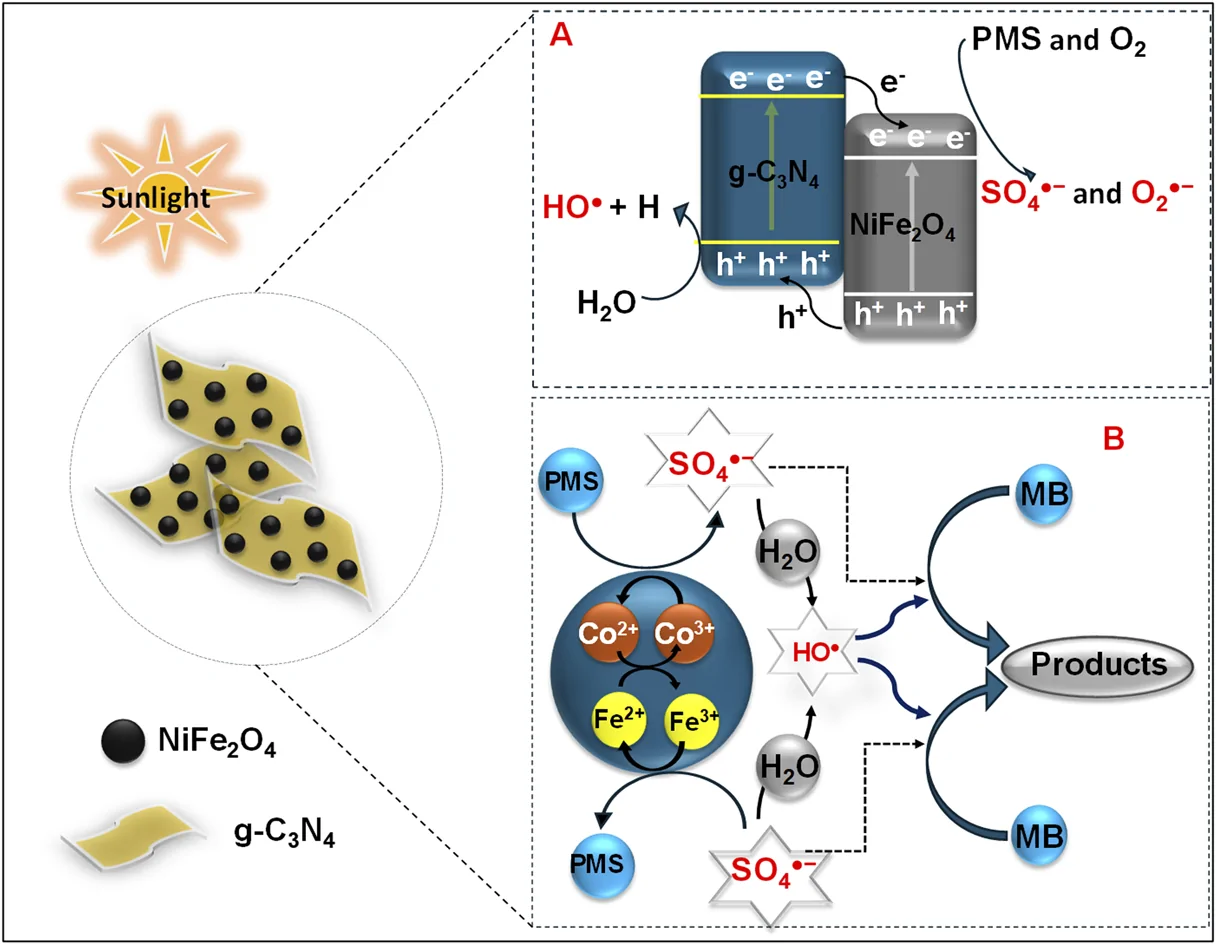

## 1 Introduction

Over the past years, high concentrations of organic compounds such as textile dyes, chlorinated compounds and pharmaceuticals have been detected in wastewater. These organic compounds are highly persistent; hence, they cannot be effectively removed from wastewater using conventional methods.^1^ Improper discharge of wastewater containing organic pollutants leads to severe health hazards and environmental pollution.^2^ Therefore, developing efficient technologies to remove organic compounds from wastewater is highly needed. Advanced oxidation processes (AOPs) such as Fenton and Fenton-like processes,^3^ electrocatalysis,^4^ catalytic ozonation,^5^ photocatalysis^6^ and persulfate oxidation^7^ have received attention due to their effectiveness in the degradation of organic pollutants.

Persulfate oxidation is a promising technology because of its high degradation efficiency and ability to function across a wide pH range.^8^ PMS is often preferred over other oxidants, such as oxygen, ozone, peroxide and persulfate, because it generates both sulfate radicals (SO_4_˙^−^) and hydroxide radicals (HO˙), whereas oxygen, ozone and peroxide generate predominantly hydroxide radicals (HO˙). Sulfate radicals have a higher reduction potential (2.5–3.1 V) than hydroxide radicals (˙OH) (1.8–2.7 V). There are several processes in which PMS can be activated to generate radical species. These processes include photocatalysis,^9^ sonocatalysis^10^ and redox reactions.^11^ PMS activation is a sluggish process due to the high activation energy required to break the bonds; hence, a good and suitable catalyst is always required. Various catalysts ranging from carbon-based to metal and metal oxide catalysts, such as graphitic carbon nitride,^12^ spinel metal ferrites,^13^ and electrospun nanofibers,^14^ have shown tremendous photocatalytic and persulfate oxidation abilities.

g-C_3_N_4_ is a prominent photocatalyst with excellent photostability, good visible-light absorption and a moderate band gap (∼2.7 eV).^15^ g-C_3_N_4_ has unique electronic properties and can be used as an electron donor to reduce the leaching of metal ions. It has been used in photocatalysis, and the photocatalytic activation of PMS has shown promising results. However, g-C_3_N_4_ suffers from a few drawbacks such as fast electro–hole pair recombination, low quantum efficiency and low surface area.^15^ To combat these drawbacks, the construction of a heterojunction through the fabrication of a composite has been employed.^16,17^ The heterojunction promotes the separation of the electron–hole pairs and increases the photocatalytic efficiency.^18^ Spinel NiFe_2_O_4_ has low solubility, a stable crystalline structure, and good magnetic properties, which facilitate catalyst recovery and tremendous persulfate oxidation properties.^19^ Spinel NiFe_2_O_4_ particles function as 0D nanoparticles; however, when utilized in conjunction with 2D nanoparticles, the interfacial contact is enhanced, subsequently facilitating the separation of charge carriers (electron–hole pairs). This phenomenon improves photocatalytic efficiency.^20^

Although a composite of NiFe_2_O_4_/g-C_3_N_4_ has been reported previously by Silva *et al.*^21^ and Palanivel *et al.*,^22^ the scope of these studies differs from the present work. Specifically, Silva *et al.* reported the utilization of NiFe_2_O_4_/g-C_3_N_4_ for peroxymonosulfate activation to degrade tetracycline hydrochloride, whereas this study focuses on methylene blue degradation. Meanwhile, Palanivel *et al.* used NiFe_2_O_4_/g-C_3_N_4_ for the degradation of methylene blue and rhodamine B, with H_2_O_2_ as the oxidant or radical initiator, whereas in this study, peroxymonosulfate was used as the radical initiator, thereby generating sulfate radicals. Alternatively, graphene oxide-encapsulated g-C_3_N_4_/ZnO ternary composites were reported for degradation of methylene blue, with a degradation efficiency of 90%.^23^ In another study, NiFe_2_O_4_ was utilized for the photocatalytic-assisted Fenton degradation of methylene blue, with a degradation efficiency of 99% after 250 min.^24^ These studies have shown that both NiFe_2_O_4_ and g-C_3_N_4_ are promising photocatalysts for methylene blue degradation. Hence, based on these studies, the construction of the 0D/2D NiFe_2_O_4_/g-C_3_N_4_ heterojunction is anticipated to improve the photocatalytic activation of PMS and the degradation efficiency of methylene blue. Methylene blue was chosen in this study because it is extensively used in the textiles, leather and pharmaceutical industries, making it one of the most common wastewater pollutants.^25^

## 2 Experimental

### 2.1 Materials

The following chemicals were purchased and used without further purification:

Nickel acetate (Ni(CH_3_COO)_2_, Sigma-Aldrich), sodium hydroxide (NaOH, Merck, 99%), dimethylformamide (99%, Merck), methylene blue (99%, Merck), peroxymonosulphate (PMS, Aldrich, 99%), hydrochloric acid (HCl, Merck, 37%), furfuryl alcohol (FFA), iron(iii) chloride hexahydrate (FeCl_3_·6HO), 4-benzoquinone (BQ, Sigma), ethanol (Merck), *tert*-butyl alcohol (TBA, Merck), and melamine (C_3_H_6_N_6_, Merck). Wastewater was collected from the Mhlathuze wastewater plant, KwaZulu Natal, South Africa; coordinates: latitude −28.76; longitude 32.07.

### 2.2 Catalysts preparation

#### 2.2.1 Synthesis of 0D g-C_3_N_4_

g-C_3_N_4_ was synthesized by thermal polymerization, as reported in the literature with a slight modification.^17^ Instead of using urea as a starting material, which was previously used,^17^ 10 g of melamine was placed in a porcelain crucible and heated to a temperature of 500 °C at a rate of 2 °C min^−1^ and maintained at this temperature for 5 h. The resultant light-yellow powder (g-C_3_N_4_ NS) was cooled and collected (Scheme 1A).

**Scheme 1:**
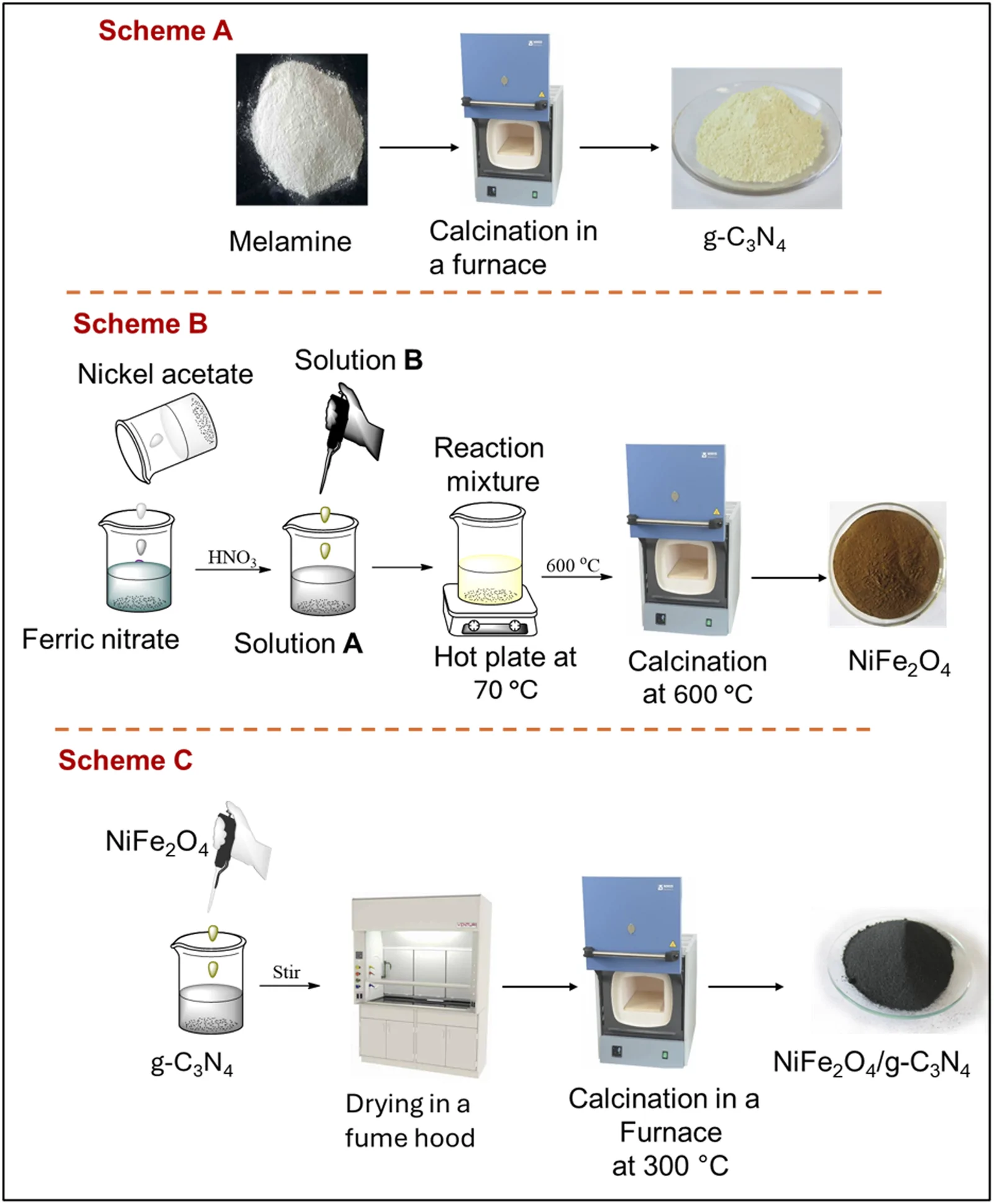
(A) Preparation of g-C_3_N_4_, (B) preparation of NiFe_2_O_4_ NPs and (C) preparation of the NiFe_2_O_4_/g-C_3_N_4_ catalyst.

#### 2.2.2 Synthesis of 0D NiFe_2_O_4_ nanoparticles

NiFe_2_O_4_ nanoparticles were synthesized as previously reported.^11^ Briefly, in a beaker, aqueous solutions of ferric nitrate (0.02 mol) and nickel acetate (0.01 mol) were mixed and consistently agitated before adding HNO_3_ (0.44 mol). The mixture was named solution A. A solution containing 0.09 moles of sugar was slowly mixed with 0.25 moles of monoethanolamine (ETA) and named solution B. Solution B was slowly transferred to solution A. The mixture was heated on a hot plate until it experienced a significant change and turned into a large, black material. The large, black precursor material was subjected to calcination at 600 °C in a muffle furnace under air flow for roughly 7 h until a brown powder was obtained,^26^ as shown in Scheme 1B. The muffle furnace was heated and stabilized at 600 °C before the precursor was subjected to heating.

#### 2.2.3 Synthesis of 0D/2D NiFe_2_O_4_/g-C_3_N_4_

Two solutions of NiFe_2_O_4_ (1 g) and g-C_3_N_4_ (10 g) were prepared by dissolving both NiFe_2_O_4_ and g-C_3_N_4_ in 5 mL of ethanol in separate containers, followed by stirring for 1 h. The solution of NiFe_2_O_4_ was slowly (dropwise) added to a solution of g-C_3_N_4_ with continuous stirring. The mixture was further stirred for 3 h. The solvent was dried in a high vacuum fume hood, followed by calcination at 300 °C in a furnace for 3 h. The NiFe_2_O_4_/g-C_3_N_4_ composite was collected and transferred to a container and stored in the dark, as shown in Scheme 1C.

### 2.3 Photocatalytic degradation studies

The degradation studies were performed using natural sunlight. The temperature, precipitation, humidity and wind were reported to be 29 °C, 5%, 68% and 24 km h^−1^, respectively. These conditions were chosen because they are more common in South Africa in summer, which would be beneficial for practical application. 5 mL of 40 mg L^−1^ methylene blue was used for all the studies. PMS (1 mL, 0.1 M) and NiFe_2_O_4_, g-C_3_N_4_ and NiFe_2_O_4_/g-C_3_N_4_ catalysts (20 mg) were used. The untreated wastewater samples were collected from the Mhlathuze wastewater plant (KwaZulu Natal, South Africa, coordinates: latitude −28.76; longitude 32.07). The water in the Mhlathuze wastewater plant is a combination of sewage, industrial effluent and domestic wastewater from households in Empangeni, Richards Bay, and Felixton. The Mhlathuze wastewater plant was chosen because of its location. Its wastewater system is generated by major industries in the region, including factories and chemical processing plants, particularly in the industrial areas of Richards Bay, Empangeni and Felixton. These samples were used for the preparation of methylene blue solutions. The studies were performed in triplicate, and the arithmetic mean was calculated. Before the degradation studies were performed, the mixture of the catalysts and the solution were kept in the dark for 60 min to reach adsorption–desorption equilibrium. Eqn (1) was used to calculate the degradation efficiency, where *C*_o_ is the initial concentration of methylene blue, and *C*_*t*_ is the concentration of methylene blue at time *t* during degradation.1
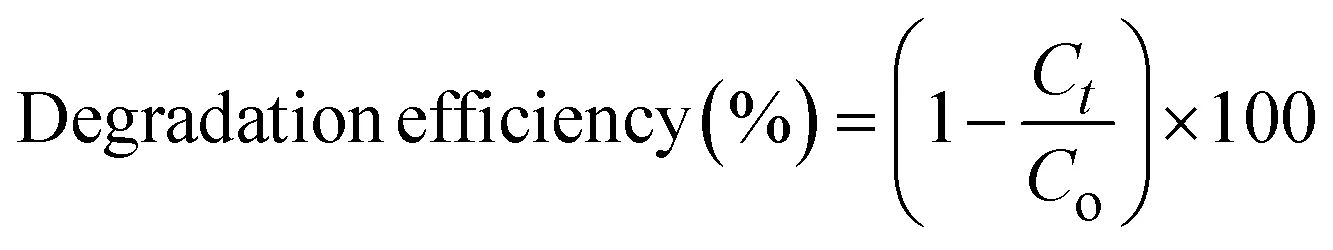


Initially, control experiments for methylene blue degradation were conducted in the presence of light alone and PMS alone. Comparative degradation studies were then performed under various combinations: PMS with light, PMS with catalysts and light with catalysts. Subsequently, methylene blue degradation studies were performed in the system combining PMS, sunlight and catalysts. The kinetic data were obtained by fitting ln(*C*_o_/*C*_*t*_) against time (min). The rate constants (*k*_obs_) were obtained from the slopes. The half-lives were calculated using the equation *t*_1/2_ = 0.693/*k*_obs_. The reaction setup is shown in Fig. S1.

### 2.4 Catalyst characterization

A Bruker Tensor 27 FT-IR spectrometer was used to acquire the FT-IR spectra and carry out the analysis with a standard ATR cell. The analysis was performed in the mid-IR region between 450 and 4000 cm^−1^.

XRD spectra were acquired using a Bruker AXS D8 diffractometer instrument equipped with monochromatic Cu Kα radiation (*λ* = 1.5406 Å) operated at 40 mA and 40 kV. The instrument was operated over a scan range of 10°–90° with a wavelength of 1.5406 Å and a scan speed of 0.3° min^−1^.

Scanning electron microscopy (SEM) imaging was performed using a Carl Zeiss FE-SEM Sigma VP-03-67 instrument. The acceleration voltage was set at 20 kV. The sample holder was a carbon tape mounted on a stub. Gold was used as a coating material.

Transmission electron microscopy (TEM), selected area electron diffraction (SAED) and high-resolution electron microscopy (HRTEM) analyses were performed using a JEOL 2100 HRTEM instrument. The analyses were performed at an accelerating voltage of 200 kV.

The absorption properties of g-C_3_N_4_, NiFe_2_O_4_ and the NiFe_2_O_4_/g-C_3_N_4_ composite were determined using a PerkinElmer Lambda 1050 NIR UV-visible spectrophotometer.

The Brunauer–Emmett–Teller (BET) analysis was performed using a Micrometrics ASAP 2020 surface area and porosity analyzer to obtain nitrogen adsorption/desorption isotherms. The samples were degassed with helium prior to analysis.

## 3 Results and discussions

### 3.1 Characterization of the catalysts

#### 3.1.1 Fourier transform infrared (FT-IR) spectroscopy

The FT-IR spectra of g-C_3_N_4_, NiFe_2_O_4_ and NiFe_2_O_4_/g-C_3_N_4_ are shown in Fig. 1. The spectrum of g-C_3_N_4_ displays an intense peak at 800 cm^−1^, corresponding to the compression modes and vibrational stretching of the triazine ring. Several peaks are observed between 1000 and 1650 cm^−1^, corresponding to the stretching vibrations of tri-*s*-triazine units. A broad peak observed at 3140 cm^−1^ corresponds to the stretching vibration of the O–H bond emanating from the surface-adsorbed water.^27,28^ The spectrum of NiFe_2_O_4_ (Fig. 1) displays a strong peak at 530 cm^−1^ attributed to the stretching vibrations of the metal oxide in the tetrahedral and octahedral sites.^29^ The spectrum of NiFe_2_O_4_/g-C_3_N_4_ (Fig. 1) displays peaks at 800 and between 1000 and 1650 cm^−1^, corresponding to g-C_3_N_4_. A weak peak is observed at 530 cm^−1^, corresponding to NiFe_2_O_4_.

**Fig. 1:**
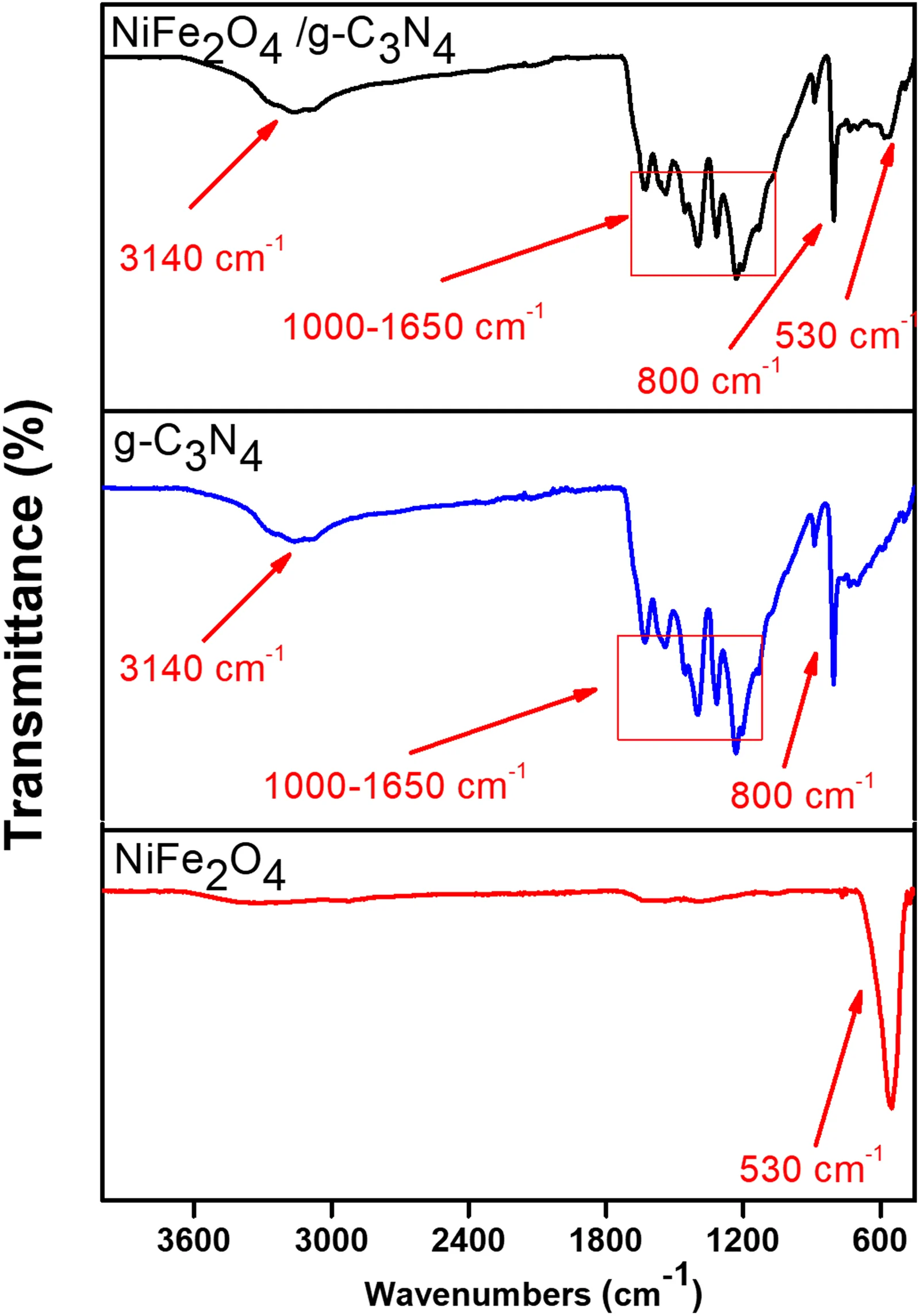
FT-IR spectra of NiFe_2_O_4_, g-C_3_N_4_ and NiFe_2_O_4_/g-C_3_N_4_.

#### 3.1.2 X-ray diffraction (XRD)

The XRD patterns of g-C_3_N_4_, NiFe_2_O_4_ and NiFe_2_O_4_/g-C_3_N_4_ are shown in Fig. 2. The XRD pattern of g-C_3_N_4_ displays a peak at 2*θ* = 27°, corresponding to the (002) Miller indices of g-C_3_N_4_.^30^ The XRD pattern of NiFe_2_O_4_ displays peaks at 2*θ* = 18.3°, 30.1°, 35.4°, 37.1°, 43.1°, 53.8°, 57.3°, and 62.6°, corresponding to (111), (220), (311), (222), (400), (422), (511), and (440) planes, respectively, of the spinel NiFe_2_O_4_.^31,32^ The XRD pattern of NiFe_2_O_4_/g-C_3_N_4_ displays an intense peak at 2*θ* = 27.2°, corresponding to the (002) Miller index, and low intensity peaks at 2*θ* = 18.3°, 30.1°, 35.4°, 37.1°, 43.1°, 53.8°, 57.3°, and 62.6°, corresponding to (111), (220), (311), (222), (400), (422), (511), and (440) planes, respectively, of the spinel NiFe_2_O_4_ are observed. This confirms the existence of both g-C_3_N_4_ and NiFe_2_O_4_. The results showed that there was no observable shift in the (002) peak corresponding to g-C_3_N_4_ upon the incorporation of NiFe_2_O_4_ NPs. The crystallite size of g-C_3_N_4_ and NiFe_2_O_4_ NPs and g-C_3_N_4_, NiFe_2_O_4_ and NiFe_2_O_4_/g-C_3_N_4_ were investigated using the Scherrer equation, eqn (2).2
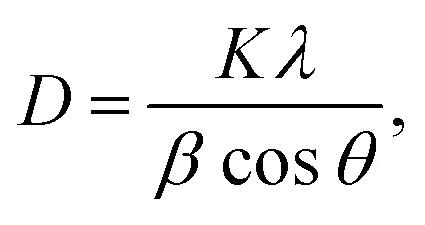
where *D* is the crystaliine size, *K* is the shape factor (*K* = 0.90), *λ* is the wavelength (*λ* = 0.15406 nm), *β* is line broadening measured as the full width at half maximum of the diffraction peak, and *θ* is Bragg's angle (corresponding to half of the experimental 2*θ* peak position). Both 2*θ* and *β* are measured in radians. The mean size of pristine g-C_3_N_4_ was calculated to be 12 nm, while the mean size of NiFe_2_O_4_ was calculated to be 29 nm. The mean size of NiFe_2_O_4_/g-C_3_N_4_ was calculated to be 17 nm.

**Fig. 2:**
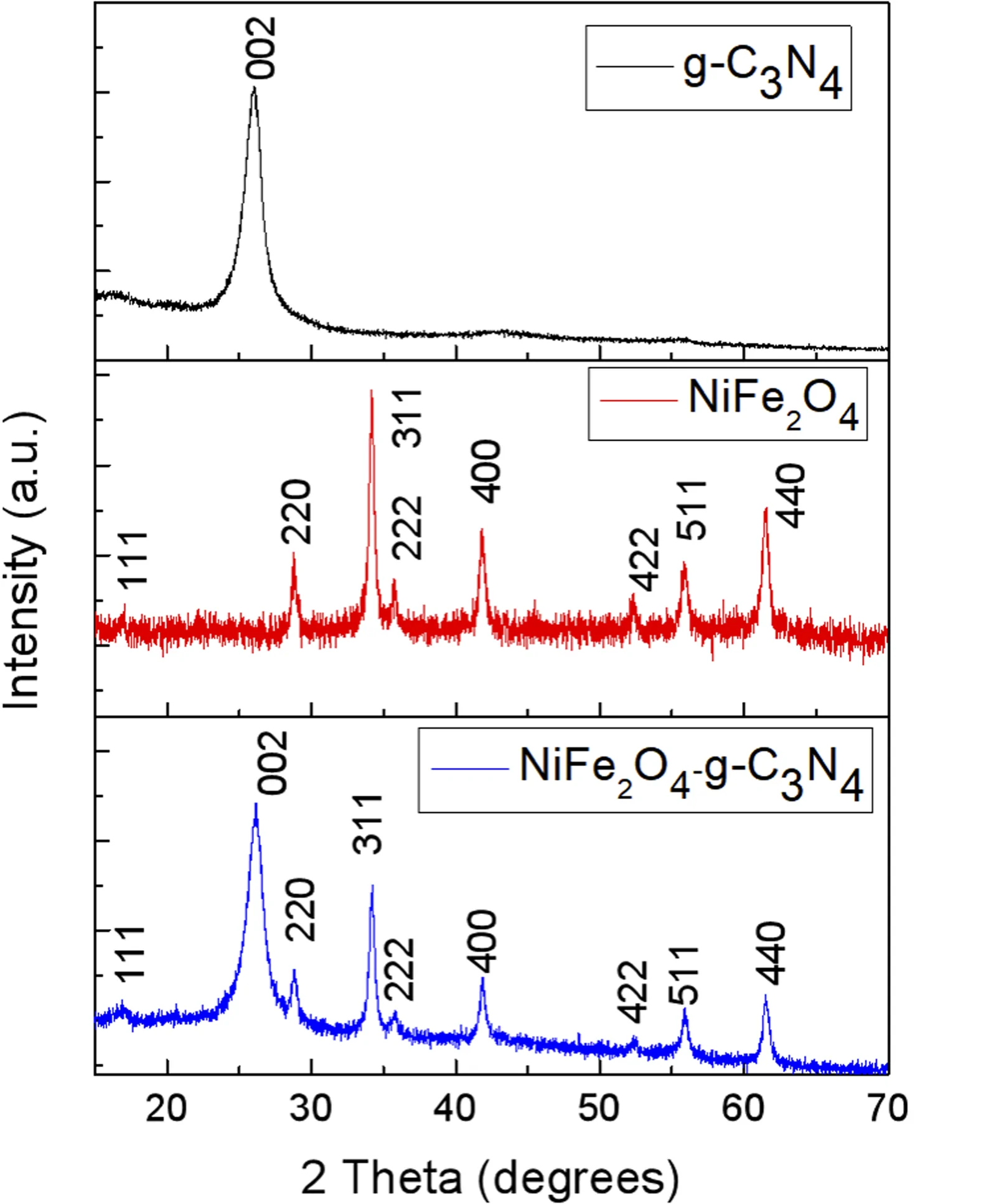
XRD patterns of NiFe_2_O_4_, g-C_3_N_4_ and NiFe_2_O_4_/g-C_3_N_4_.

However, the Scherrer method only provides information regarding crystal size and ignores internal lattice strain. Hence, the William–Hall method was utilized to investigate the crystal size and microstrain. The William–Hall equation is shown in eqn (3).3
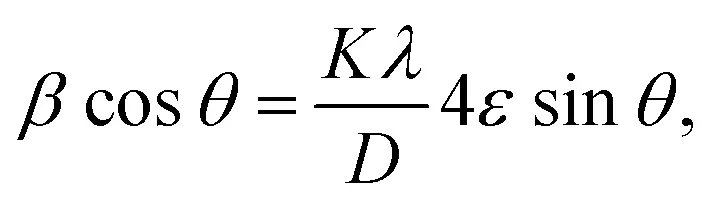
where *ε* is the effective microstrain. The equation represents a straight line in which *ε* is the slope. The plot of NiFe_2_O_4_/g-C_3_N_4_ is shown in Fig. S5 as an example. The mean crystalline sizes of g-C_3_N_4_, NiFe_2_O_4_ and NiFe_2_O_4_/g-C_3_N_4_ were 16 nm, 31 nm and 23 nm, respectively. The mean crystalline sizes obtained from the Williamson–Hall method were slightly higher than those obtained from the Scherrer method. This is attributed to the fact that the Scherrer method assumes that peak broadening arises solely from finite crystal size, whereas the William–Hall method decouples the additional peak broadening contributions from crystal defects and lattice strain.

The lattice strains calculated for g-C_3_N_4_, NiFe_2_O_4_ and NiFe_2_O_4_/g-C_3_N_4_ were 0.000642, 0.000873 and 0.00112, respectively. The lattice strain of the NiFe_2_O_4_/g-C_3_N_4_ composite was higher than those obtained from pristine g-C_3_N_4_ and NiFe_2_O_4_, suggesting that the composite has a high density of defect sites.

#### 3.1.3 Raman spectroscopy

Raman spectroscopy was performed to further investigate the interaction between g-C_3_N_4_ and NiFe_2_O_4_. The Raman spectra of g-C_3_N_4_, NiFe_2_O_4_ and NiFe_2_O_4_/g-C_3_N_4_ are displayed in Fig. 3. g-C_3_N_4_ displays the anticipated peaks at 1280 cm^−1^ and 1600 cm^−1^ attributed to the D-band (sp^3^) and G-band (sp^2^), respectively.^33^ The Raman spectrum of NiFe_2_O_4_ displays peaks corresponding to A_1g_, E_g_ and T_2g_ mode. The peak for the A_1g_ mode is due to the stretching of oxygen atoms in the tetrahedral sites of the Fe–O bonds. The peak for the E_g_ mode was attributed to the bending of oxygen with respect to the metal ion, while the peak for the T_2g_ mode was assigned to the asymmetric oxygen bond bending. The spectrum of NiFe_2_O_4_/g-C_3_N_4_ displays peaks associated with g-C_3_N_4_ and NiFe_2_O_4_. However, the formation of the composite induced a shift in the D and G bands of g-C_3_N_4_ to higher frequencies. This is believed to have emanated from the strong interactions between g-C_3_N_4_ and NiFe_2_O_4_. The *I*_D_/*I*_G_ ratios calculated for g-C_3_N_4_ and NiFe_2_O_4_/g-C_3_N_4_ were 0.27 and 0.96, respectively. An apparent increase in the *I*_D_/*I*_G_ ratio in NiFe_2_O_4_/g-C_3_N_4_ was caused by defect sites in the NiFe_2_O_4_/g-C_3_N_4_composite compared to g-C_3_N_4_ due to the strong interaction between g-C_3_N_4_ and NiFe_2_O_4_.^22^

**Fig. 3:**
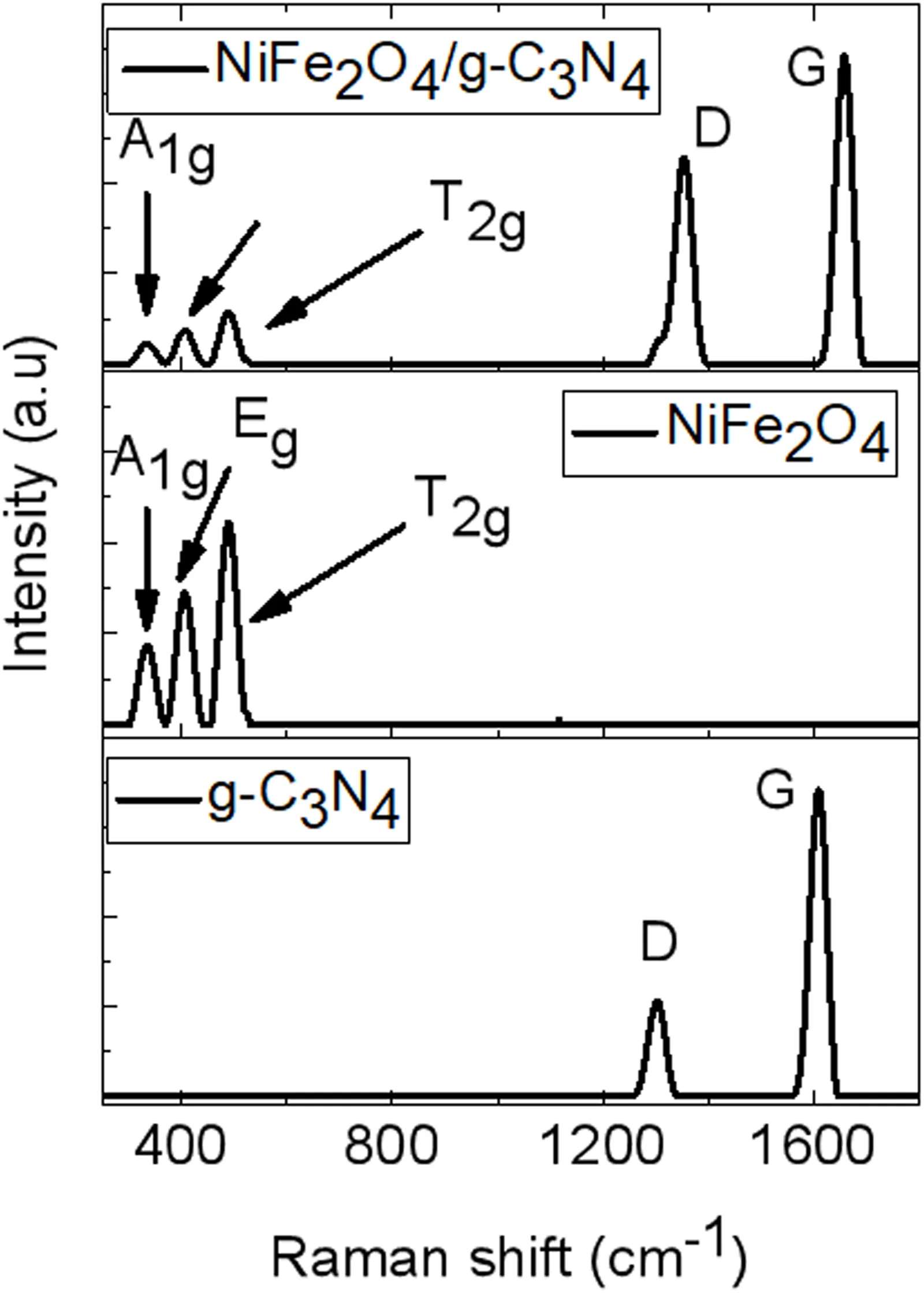
Raman spectra of g-C_3_N_4_, NiFe_2_O_4_ and NiFe_2_O_4_/g-C_3_N_4_.

#### 3.1.4 Brunauer–Emmett–Teller surface analysis

The BET surface analysis was carried out to determine the surface areas, pore volumes and pore diameters of the g-C_3_N_4_, NiFe_2_O_4_ and NiFe_2_O_4_/g-C_3_N_4_ catalysts. The BET isotherms are shown in Fig. 4. All the catalysts displayed properties of a type IV material with a hysteresis loop. This suggests that the materials have mesopores.^34^ The surface areas of the g-C_3_N_4_, NiFe_2_O_4_ and NiFe_2_O_4_/g-C_3_N_4_ catalysts were 30.4 m^2^ g^−1^, 26.0 m^2^ g^−1^ and 37.4 m^2^ g^−1^, respectively. The results suggests that the NiFe_2_O_4_/g-C_3_N_4_ composite had the highest surface area, whereas the NiFe_2_O_4_ nanoparticles had the lowest surface area. The pore volumes for g-C_3_N_4_, NiFe_2_O_4_ and NiFe_2_O_4_/g-C_3_N_4_ catalysts were 0.347 cm^3^ g^−1^, 0.264 cm^3^ g^−1^ and 0.608 cm^3^ g^−1^, respectively. The pore volume of the NiFe_2_O_4_/g-C_3_N_4_ composite was higher than those of g-C_3_N_4_ and NiFe_2_O_4_. The BET surface areas and pore volumes imply that the porous structure of the catalysts is characterized by micropores. The pore widths were 29 nm, 21 nm and 27 nm for g-C_3_N_4_, NiFe_2_O_4_ and NiFe_2_O_4_/g-C_3_N_4_ catalysts, respectively. The pore width of the g NiFe_2_O_4_/g-C_3_N_4_ catalysts is lower than those of g-C_3_N_4_ and NiFe_2_O_4_. The NiFe_2_O_4_ are hosted within the g-C_3_N_4_ pores.

**Fig. 4:**
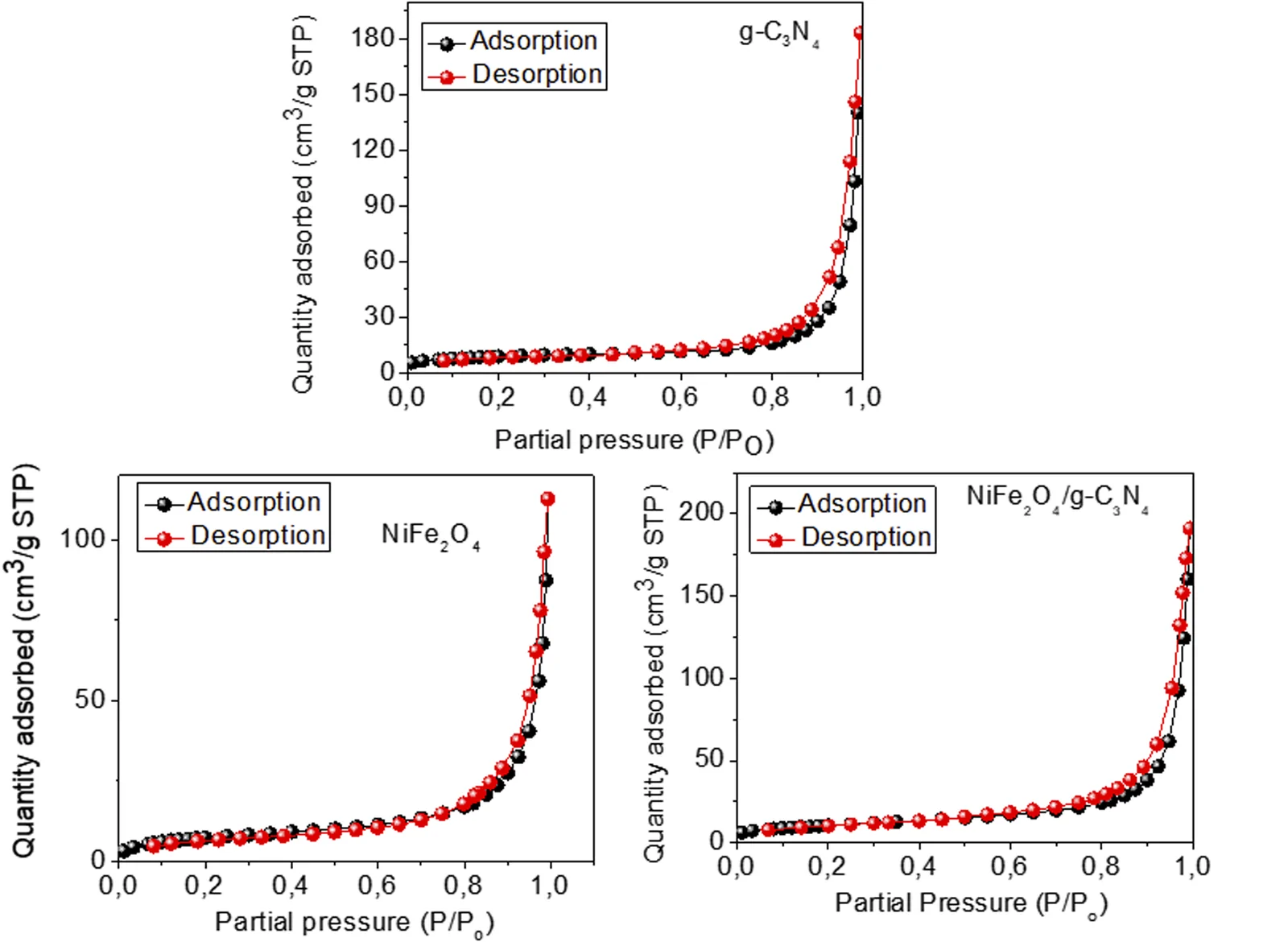
BET isotherms of g-C_3_N_4_, NiFe_2_O_4_ and the NiFe_2_O_4_/g-C_3_N_4_ composite.

#### 3.1.5 Scanning electron microscopy-energy dispersive X-ray spectroscopy (SEM-EDX) elemental mapping

SEM was performed to study the morphology and topography of the catalysts, while EDX elemental mapping was performed to confirm the elemental composition and distribution of the catalysts. The SEM images of g-C_3_N_4_, NiFe_2_O_4_ and NiFe_2_O_4_/g-C_3_N_4_ (Fig. 5A) show characteristics of a rough material with a poorly defined structure. The SEM image of g-C_3_N_4_ revealed a highly porous structure, which is ideal for the subsequent immobilization of active nanoparticles. The SEM image of NiFe_2_O_4_ shows a particular morphology characterized by agglomerated nanoparticles sounds perfect. The SEM image of NiFe_2_O_4_/g-C_3_N_4_ revealed that NiFe_2_O_4_ occupies the pores in g-C_3_N_4_, resulting in a less porous surface compared to that of g-C_3_N_4_. The SEM results further revealed that NiFe_2_O_4_ is homogeneously dispersed on g-C_3_N_4_ without significantly altering the g-C_3_N_4_ structure.

**Fig. 5:**
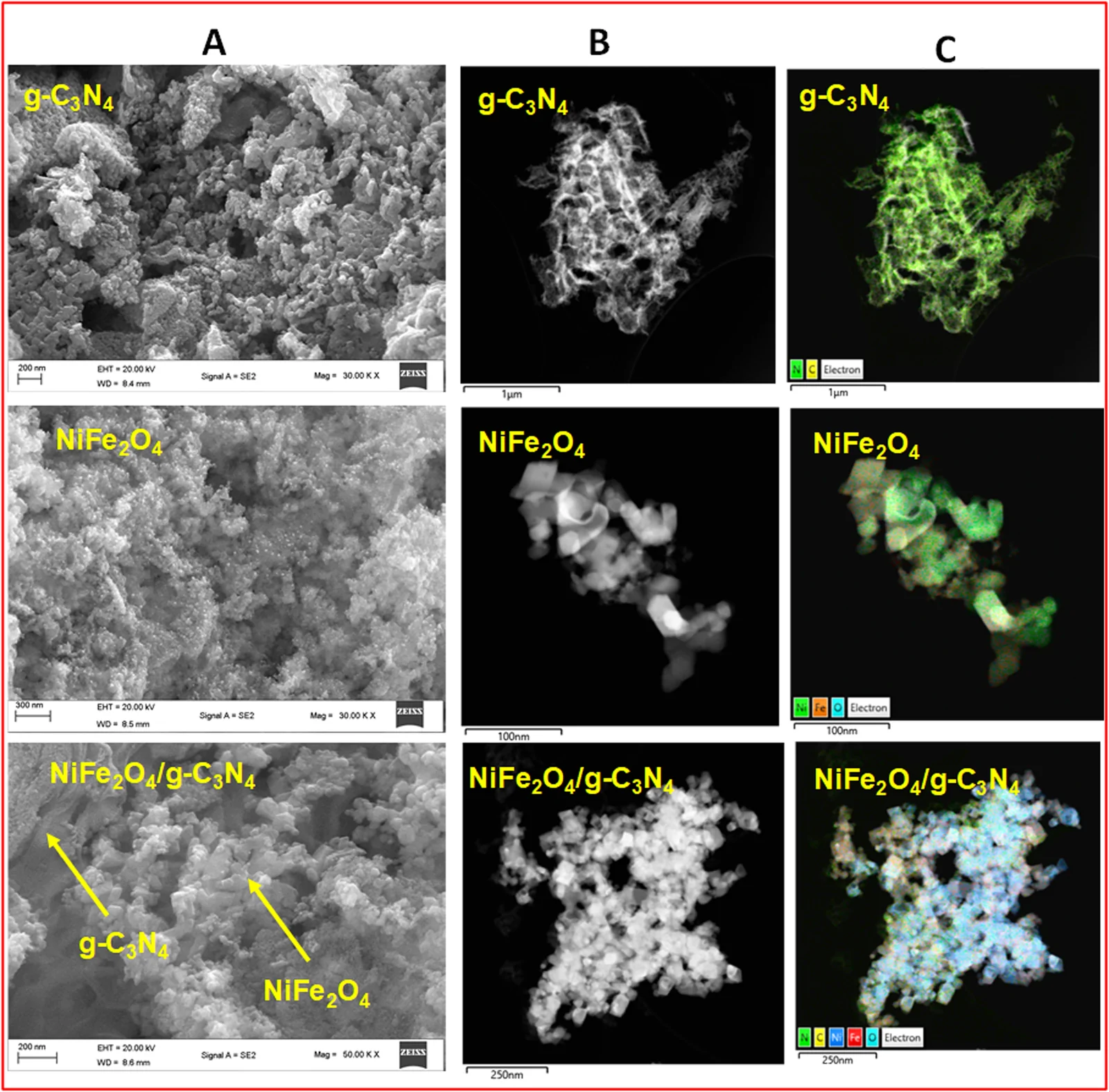
SEM images (A) EDX electron microscope images (B) and the corresponding EDX elemental mappings (C) of g-C_3_N_4_, NiFe_2_O_4_ and NiFe_2_O_4_/g-C_3_N_4_.

The EDX electron images of g-C_3_N_4_, NiFe_2_O_4_ and NiFe_2_O_4_/g-C_3_N_4_ are shown in Fig. 5B, and their corresponding elemental mapping images are shown in Fig. 5C. The EDS elemental mapping of g-C_3_N_4_ shows the presence of carbon (C) and nitrogen (N) as anticipated. The EDS elemental mapping of NiFe_2_O_4_ reveals the presence of nickel (Ni), iron (Fe) and oxygen (O) elements, which are homogeneously distributed. As expected, the EDS elemental mapping of NiFe_2_O_4_/g-C_3_N_4_ shows the presence of C, N, Ni, Fe and O. Hence, the presence of all the elements was confirmed using EDS elemental mapping analysis.

#### 3.1.6 Atomic force microscopy (AFM)

AFM was utilised to study the surface morphology and roughness of g-C_3_N_4_, NiFe_2_O_4_ and NiFe_2_O_4_/g-C_3_N_4_. The topographical data provide information on texture, roughness and structural changes, which are very important in photocatalysis. The two-dimensional and three-dimensional AFM topographic images of g-C_3_N_4_, NiFe_2_O_4_ and NiFe_2_O_4_/g-C_3_N_4_ are shown in Fig. 6. The 2D topographic image of g-C_3_N_4_ shows a smooth surface without visible particles, as shown in Fig. 6A. The 3D topographic image also shows a smooth surface with low peak-to-valley contrast. The root mean square (RMS) value was found to be 2.75 nm, as shown in Fig. 6A. This is evidence of a higher-order conjugated microporous polymer material. The AFM 2D topographic image of NiFe_2_O_4_ shows a relatively rough surface with visible particles, as shown in Fig. 6B. The 3D topographic image shows a rough surface with high peak-to-valley contrast, as shown in Fig. 6B. The obtained RMS was 12.3 nm, which is higher than that obtained in g-C_3_N_4_. The 2D topographic image of NiFe_2_O_4_/g-C_3_N_4_ displayed a rough surface with visible particles (Fig. 6C). The 3D structure also displayed a rough surface with high peak-to-valley contrast. The obtained RMS was 7.8 nm, which is higher than that obtained for pristine g-C_3_N_4_, as shown in Fig. 6C. This suggests that the fabrication of the composite resulted in an increase in surface roughness. An enhanced surface roughness of the nanomaterials in photocatalysis significantly increases the available active surface area, improves the light-harvesting ability through multiple scattering, and accelerates the turbulent mass transfer of pollutants.^35^

**Fig. 6:**
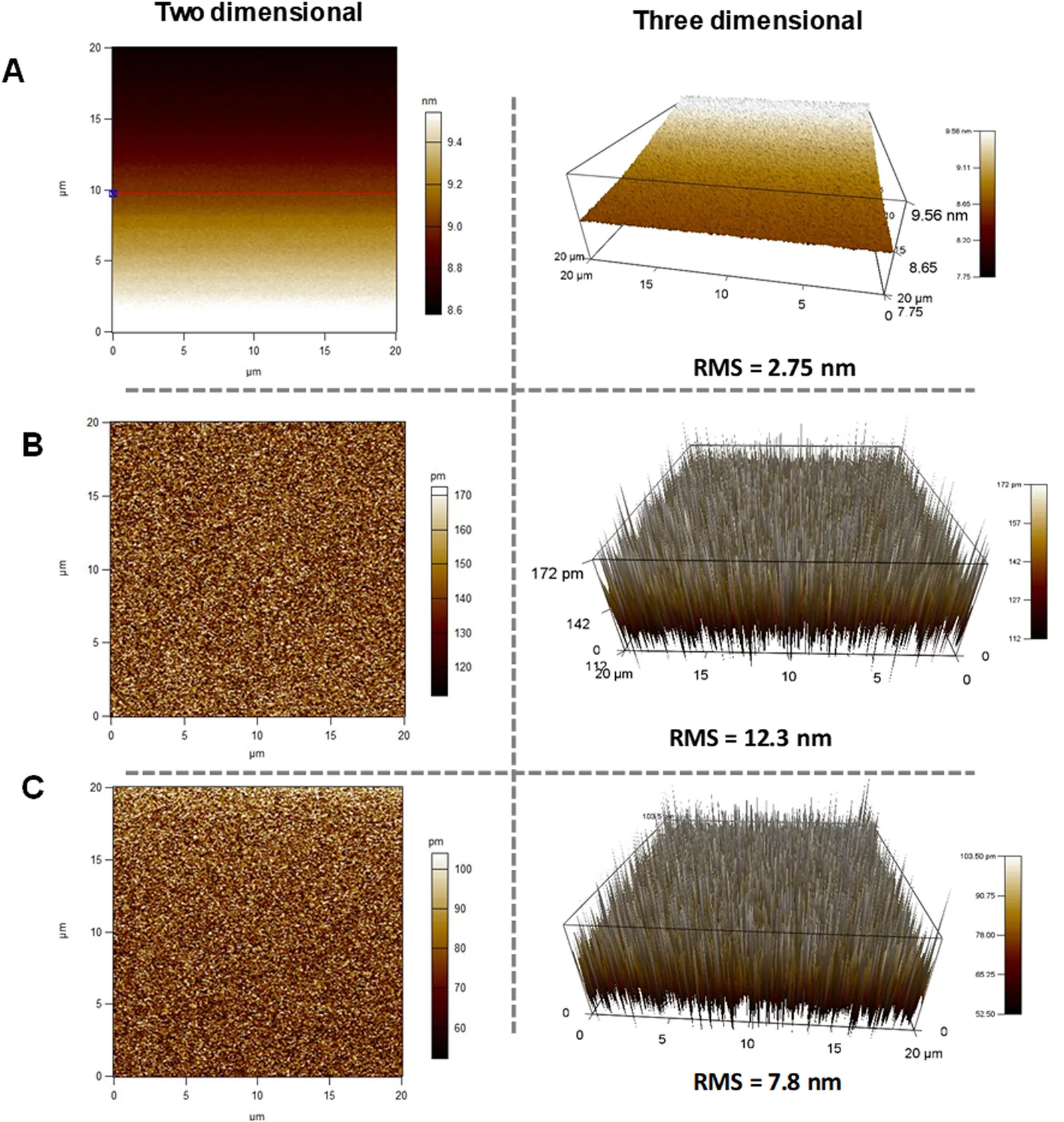
Two-dimensional and three-dimensional topographic images of the g-C_3_N_4_ (A), NiFe_2_O_4_ (B) and NiFe_2_O_4_/g-C_3_N_4_ (C) catalysts.

#### 3.1.7 Transmission electron microscopy (TEM), high-resolution transmission electron microscopy (HRTEM) and selected area electron diffraction (SAED) patterns

TEM was used to study the morphologies of the g-C_3_N_4_, NiFe_2_O_4_ and NiFe_2_O_4_/g-C_3_N_4_ catalysts. The TEM images of g-C_3_N_4_, NiFe_2_O_4_ and NiFe_2_O_4_/g-C_3_N_4_ catalysts are shown in Fig. 7A. The TEM images of NiFe_2_O_4_ and g-C_3_N_4_ display two-dimensional structures. The TEM image of g-C_3_N_4_ displays the properties of highly porous, elongated 2D nanosheets. The TEM image of the NiFe_2_O_4_/g-C_3_N_4_ composite shows that 2D NiFe_2_O_4_ nanoparticles are homogeneously distributed on the surface of 2D g-C_3_N_4_ to form NiFe_2_O_4_/g-C_3_N_4_.

**Fig. 7:**
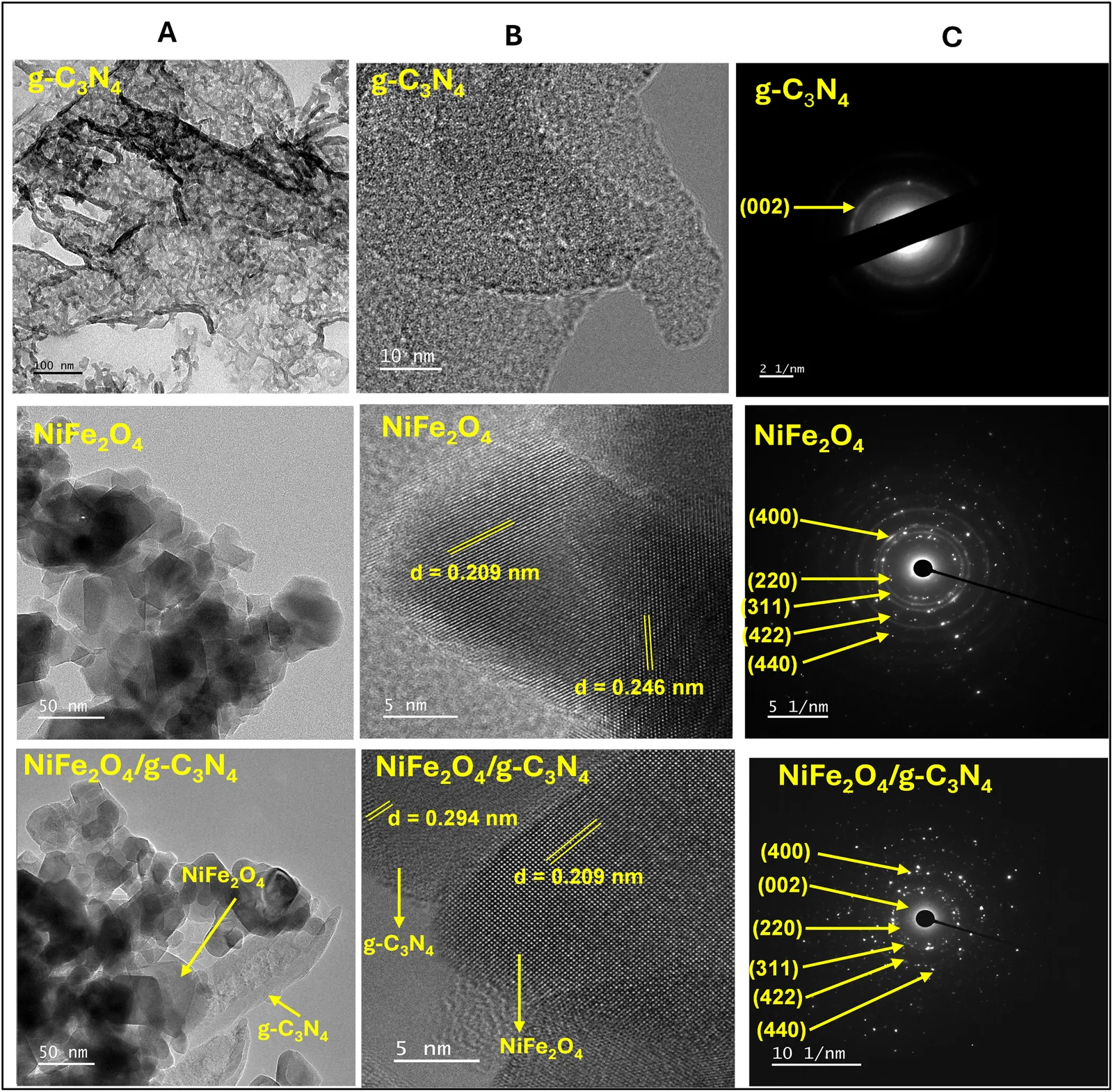
TEM images (A), HRTEM images (B) and SAED patterns (C) of g-C_3_N_4_, NiFe_2_O_4_ and NiFe_2_O_4_/g-C_3_N_4_.

HRTEM was used to study the atomic arrangements of the g-C_3_N_4_, NiFe_2_O_4_ and NiFe_2_O_4_/g-C_3_N_4_ catalysts. The HRTEM images of NiFe_2_O_4_, g-C_3_N_4_ and NiFe_2_O_4_/g-C_3_N_4_ catalysts are shown in Fig. 7B. The HRTEM image of NiFe_2_O_4_ shows lattice fringes with *d*-spacings of 0.246 nm and 0.209 nm, which correspond to the (311) and (400) Miller indices, respectively.^36^ The HRTEM image of g-C_3_N_4_ did not show any *d*-spacing, which suggests that the material is amorphous. This is in correlation with the XRD results, which showed a broad diffraction peak corresponding to the (002) Miller index. The HRTEM image of NiFe_2_O_4_/g-C_3_N_4_ shows lattice fringes with a *d*-spacing of 0.209 nm for the (400) Miller index of NiFe_2_O_4_. Another lattice fringe with *d*-spacing of 0.294 nm was observed, which corresponds to the (220) lattice plane of NiFe_2_O_4_ .^37^ From the HRTEM image of NiFe_2_O_4_/g-C_3_N_4_, the interface between NiFe_2_O_4_ and g-C_3_N_4_ was perceived.

The selected area electron diffraction (SAED) patterns for NiFe_2_O_4_, g-C_3_N_4_ and NiFe_2_O_4_/g-C_3_N_4_ catalysts are shown in Fig. 7C. The pattern of NiFe_2_O_4_ shows diffraction rings corresponding to (111), (220), (311), (400), (422) and (440) planes.^32^ The pattern of g-C_3_N_4_ shows only one ring corresponding to the (002) Miller index. The SAED pattern of NiFe_2_O_4_/g-C_3_N_4_ shows diffraction rings corresponding to (111), (220), (311), (400), (422) and (440) planes of NiFe_2_O_4_ and an additional diffraction ring corresponding to the (002) Miller index of g-C_3_N_4_. This confirms that both NiFe_2_O_4_ and g-C_3_N_4_ are present in the NiFe_2_O_4_/g-C_3_N_4_ composite.

## 4 Degradation studies

### 4.1 Spectral changes

Prior to irradiation, the mixture was kept in the dark for 30 min while monitoring adsorption using UV-vis spectroscopy at 15 min intervals. The UV-vis spectra are shown in Fig. S3. The results suggest that the adsorption–desorption equilibrium was achieved after 45 min. The degradation of methylene blue was evaluated by assessing the UV-vis spectral changes at 665 nm.^38^ The aliquots were collected and analysed at 15 min intervals for 90 min using UV-vis spectroscopy. When methylene blue degradation was performed in the presence of PMS alone, light alone and PMS combined with light, no spectral changes were observed. These results suggest that no methylene blue degradation occurred under these conditions. When degradation studies were performed in the presence of light combined with catalysts (photocatalytic process), PMS combined with catalysts (catalytic PMS activation), and PMS combined with light and catalysts (photocatalytic PMS activation), methylene blue spectral changes were recorded, which suggested that degradation occurred. The colour fading of the methylene blue solution can be observed. The UV-vis spectral changes of methylene blue recorded at 15 min intervals during degradation using NiFe_2_O_4_, g-C_3_N_4_ and NiFe_2_O_4_/g-C_3_N_4_ in the presence of PMS and sunlight are shown in Fig. 8a–c. The images of the methylene blue solutions (aliquots) collected from the reaction mixture prior to degradation and after 90 min of degradation in the presence of sunlight and PMS using NiFe_2_O_4_/g-C_3_N_4_ catalyst are displayed in Fig. 8d. The FT-IR results obtained before and after methylene blue degradation are shown in Fig. S4. The spectra of the reaction mixture after 60 min of degradation and after 90 min of degradation reveal the presence of C=O peaks, which originate form intermediate degrdation product. Ultimately,CO_2_ is the final product of methylene blue degradation.

**Fig. 8:**
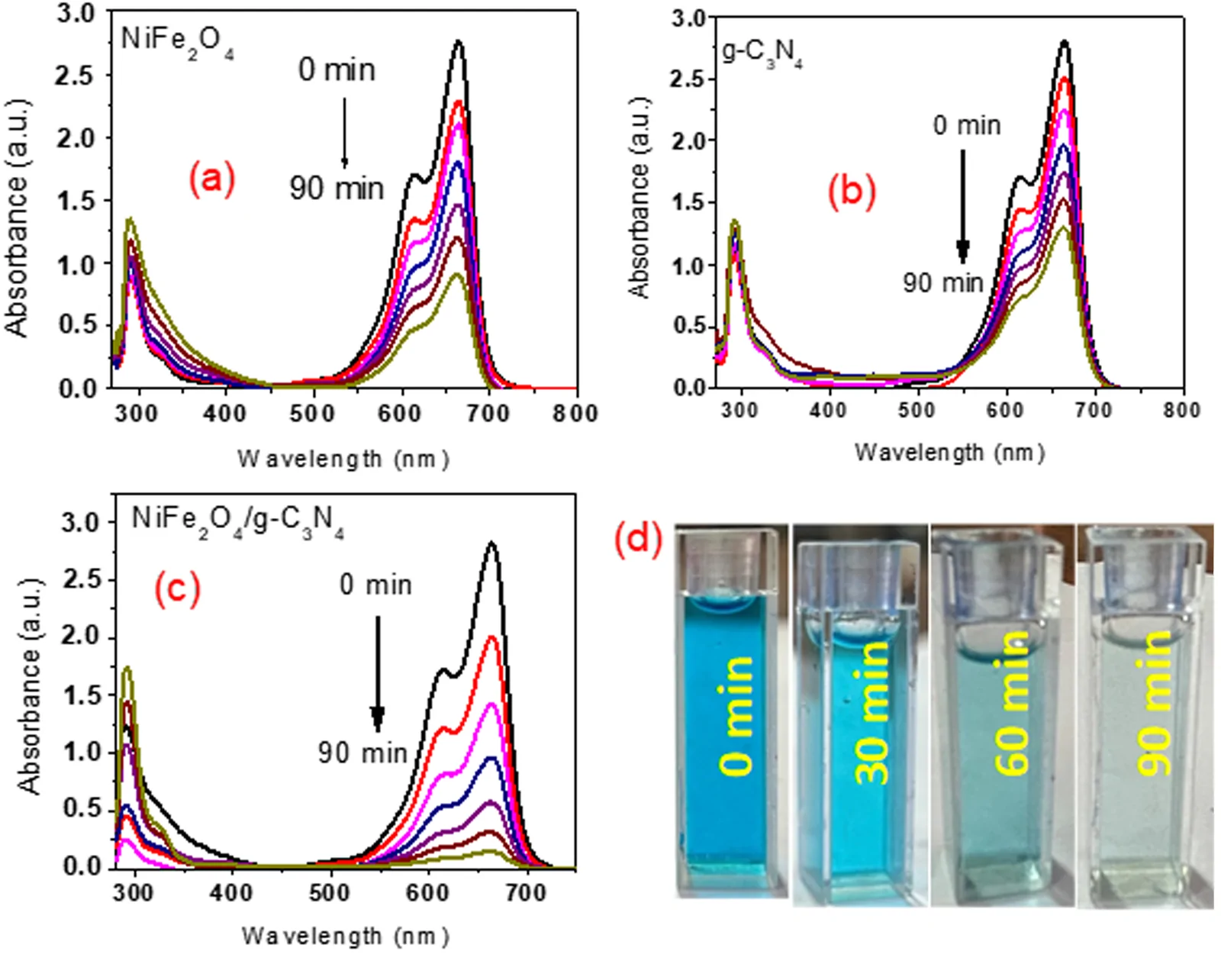
Methylene blue spectral changes observed during methylene blue degradation using the NiFe_2_O_4_, g-C_3_N_4_ and NiFe_2_O_4_/g-C_3_N_4_ catalysts in the presence of PMS (a)–(c), and images of the methylene blue solutions (aliquots) collected from the reaction mixture prior to degradation and at various time intervals (d).

### 4.2 Catalytic performance

The results for methylene blue degradation using the NiFe_2_O_4_, g-C_3_N_4_ and NiFe_2_O_4_/g-C_3_N_4_ catalysts in the presence of PMS (PMS + catalyst) are shown in Fig. 9a and Table 1. When methylene blue degradation studies were performed in the presence of PMS using the NiFe_2_O_4_, g-C_3_N_4_ and NiFe_2_O_4_/g-C_3_N_4_ catalysts, degradation efficiencies of 44%, 19% and 74%, respectively, were obtained.

**Fig. 9:**
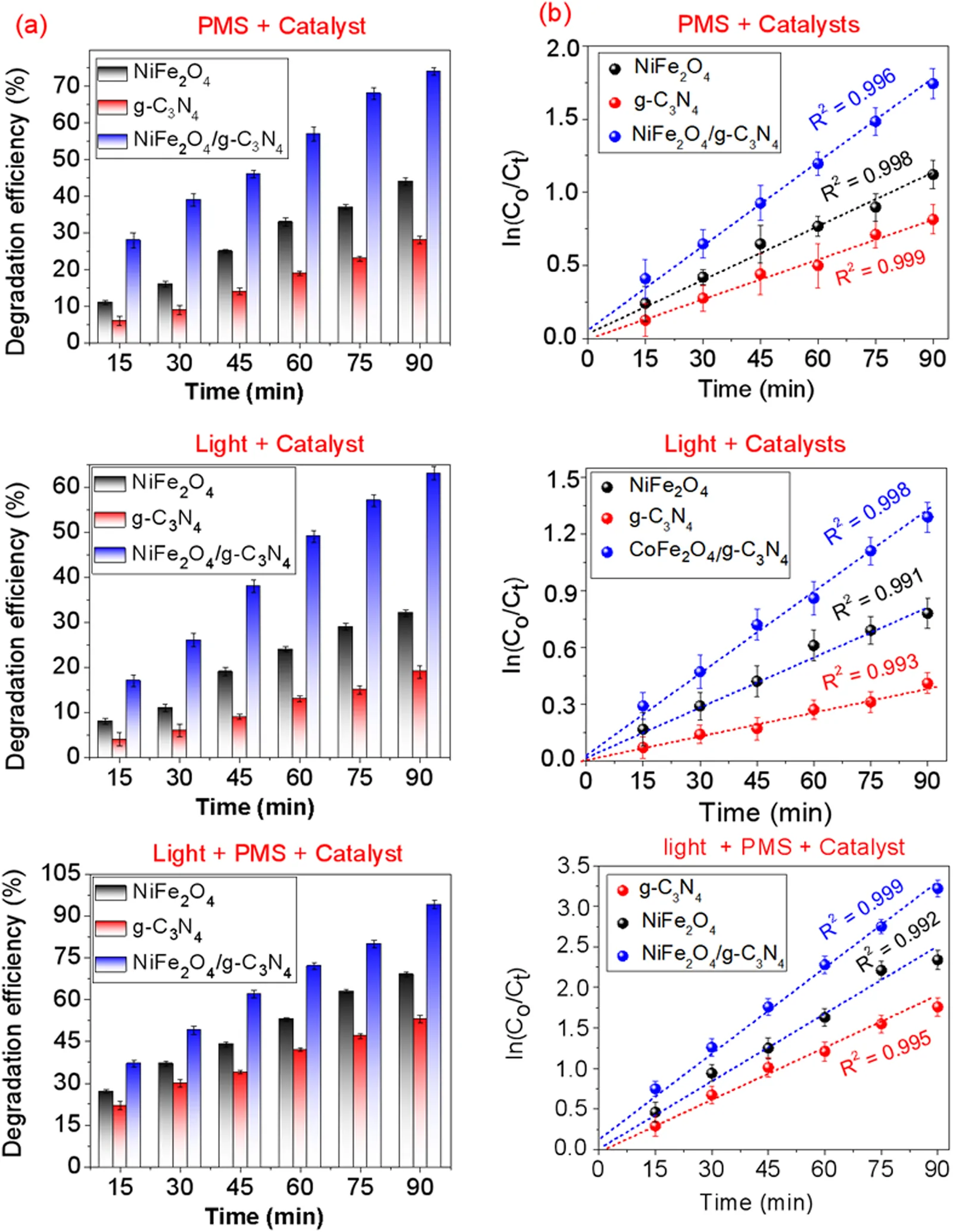
Methylene blue degradation efficiencies (a) and kinetics (b) in the presence of PMS + catalyst (PMS activation), light + catalyst (photocatalysis) and PMS + light + catalyst (photocatalytic PMS activation) using the NiFe_2_O_4_, g-C_3_N_4_ and NiFe_2_O_4_/g-C_3_N_4_ catalysts.

**Table 1:** Catalytic degradation efficiencies and reaction kinetics for methylene blue degradation using the NiFe_2_O_4_, g-C_3_N_4_ and NiFe_2_O_4_/g-C_3_N_4_ catalysts^a^

Catalyst	Degradation efficiency (%)			*k*_obs_ (min^−1^)			*τ*_1/2_ (min)		
	PMS + cat	Light + cat	PMS + light + cat	PMS + cat	Light + cat	PMS + light + cat	PMS + cat	Light + cat	PMS + light + cat
NiFe_2_O_4_	44	32	69	0.016	0.012	0.025	43	57	28
g-C_3_N_4_	19	28	53	0.005	0.008	0.019	138	87	37
NiFe_2_O_4_/g-C_3_N_4_	74	63	97	0.028	0.021	0.034	25	33	20

a Cat = catalyst.

NiFe_2_O_4_ is more catalytically active than g-C_3_N_4_. This is because NiFe_2_O_4_ has transition metals that easily undergo redox reactions due to their multiple oxidation states. In contrast, g-C_3_N_4_ requires light to activate PMS.^21^ The NiFe_2_O_4_/g-C_3_N_4_ composite showed higher catalytic activity than both NiFe_2_O_4_ and g-C_3_N_4_. This is due to the synergic effect between NiFe_2_O_4_ and g-C_3_N_4_, which promotes the activation of PMS.

When degradation of methylene blue was performed using the NiFe_2_O_4_, g-C_3_N_4_ and NiFe_2_O_4_/g-C_3_N_4_ catalysts in the presence of sunlight (light + catalyst), degradation efficiencies of 32%, 28% and 63%, respectively, were obtained, as shown in Fig. 9a and Table 1. This suggests that NiFe_2_O_4_ is more active than g-C_3_N_4_ in the photocatalytic degradation of methylene blue. Generally, g-C_3_N_4_ exhibits lower photocatalytic activity than NiFe_2_O_4_ due to the rapid recombination of the photogenerated electron–hole pairs. In contrast, NiFe_2_O_4_ has a more favourable band structure and superior charge separation compared to g-C_3_N_4_, resulting in a higher degradation efficiency.^22^ Notably, the NiFe_2_O_4_/g-C_3_N_4_ composite outperformed both NiFe_2_O_4_ and g-C_3_N_4_. This was attributed to the fact that the NiFe_2_O_4_/g-C_3_N_4_ composite exhibits improved light absorption and enhanced electron–hole separation due to the formation of a heterojunction at the interface between NiFe_2_O_4_ and g-C_3_N_4_.

The methylene blue degradation efficiencies obtained from the PMS activation process using the NiFe_2_O_4_ and NiFe_2_O_4_/g-C_3_N_4_ catalysts were higher than those obtained in the photocatalytic process using similar catalysts. This is because of the presence of NiFe_2_O_4_, which can act as a redox catalyst during PMS activation. However, when g-C_3_N_4_ was used, the photocatalytic process was more effective for the degradation of methylene blue than the PMS activation process. This is because g-C_3_N_4_ is a poor redox catalyst, although it can absorb sunlight to activate PMS.^39,40^ When degradation of methylene blue was performed using the NiFe_2_O_4_, g-C_3_N_4_ and NiFe_2_O_4_/g-C_3_N_4_ catalysts in the presence of sunlight and PMS (PMS + light + catalyst), degradation efficiencies of 69%, 53% and 97%, respectively, were obtained, as shown in Fig. 9a and Table 1. Similarly, NiFe_2_O_4_ was more catalytically active than g-C_3_N_4_. This is because NiFe_2_O_4_ can activate PMS through the redox pathway and light absorption pathway, in which NiFe_2_O_4_ is first excited by sunlight, followed by the activation of PMS by the photo-excited electrons. In contrast, g-C_3_N_4_ does not easily undergo a redox reaction to activate PMS; instead, it is first excited by sunlight, followed by the activation of PMS by photo-excited electrons.^41^ The kinetics results for the PMS activation process (Fig. 9b and Table 1) using the NiFe_2_O_4_, g-C_3_N_4_ and NiFe_2_O_4_/g-C_3_N_4_ catalysts show that NiFe_2_O_4_/g-C_3_N_4_ had a higher *k*_obs_ value than those of the NiFe_2_O_4_ and g-C_3_N_4_ catalysts. This was expected since NiFe_2_O_4_/g-C_3_N_4_ was more catalytically active than NiFe_2_O_4_ and g-C_3_N_4_ catalysts. The half-life (*t*_1/2_) was shorter for NiFe_2_O_4_/g-C_3_N_4_ than those observed for the NiFe_2_O_4_ and g-C_3_N_4_ catalysts during methylene blue degradation. The kinetics results for the photocatalytic process (Fig. 9b and Table 1) using the NiFe_2_O_4_, g-C_3_N_4_ and NiFe_2_O_4_/g-C_3_N_4_ catalysts show that NiFe_2_O_4_/g-C_3_N_4_ had a higher *k*_obs_ value than those of NiFe_2_O_4_ and g-C_3_N_4_ catalysts, and the half-life was shorter for the NiFe_2_O_4_/g-C_3_N_4_ catalyst. The *k*_obs_ values obtained from the PMS activation process using NiFe_2_O_4_ and NiFe_2_O_4_/g-C_3_N_4_ were higher than those obtained from the photocatalytic process using similar catalysts. This is because NiFe_2_O_4_ and NiFe_2_O_4_/g-C_3_N_4_ exhibited higher methylene blue degradation efficiencies in the PMS activation process than in the photocatalytic process. However, the *k*_obs_ value obtained using the g-C_3_N_4_ catalyst was higher and the half-life was lower in the photocatalytic process than in the PMS activation process. This was anticipated since the photocatalytic process was more active than the PMS activation process in the presence of g-C_3_N_4_. Moreover, the use of a 0D/2D hybrid compared to a 0D/0D hybrid creates a large contact area, speeds up electron movement, stops charge loss, and captures more light, thereby increasing efficiency.^42^

The kinetics results for the photocatalytic PMS activation process (Fig. 9b and Table 1) using the NiFe_2_O_4_, g-C_3_N_4_ and NiFe_2_O_4_/g-C_3_N_4_ catalysts revealed that NiFe_2_O_4_/g-C_3_N_4_ had a higher *k*_obs_ value than those of the NiFe_2_O_4_ and g-C_3_N_4_ catalysts, and the half-life was shorter for the NiFe_2_O_4_/g-C_3_N_4_ catalyst. The *k*_obs_ obtained from the photocatalytic PMS activation process were higher than those obtained from the photocatalytic and PMS activation processes, and *t*_1/2_ was shorter for all the catalysts. This was expected since the photocatalytic PMS activation process outperformed the photocatalytic and PMS activation processes.

## 5 Effect of various parameters on the degradation of methylene blue

### 5.1 Effect of the initial concentration of methylene blue

The degradation efficiency of organic pollutants is also affected by the initial concentration of the organic pollutants.^43^ The effect of the methylene blue concentration was studied using 80 mg L^−1^, 120 mg L^−1^ and 160 mg L^−1^ in the presence of NiFe_2_O_4_/g-C_3_N_4_ and PMS under sunlight irradiation. The results were then compared with those obtained with 40 mg L^−1^ methylene blue concentration. The methylene blue degradation results are displayed in Fig. 10a. The methylene blue degradation efficiencies achieved using 40 mg L^−1^, 80 mg L^−1^, 120 mg L^−1^ and 160 mg L^−1^ were 97%, 86%, 81% and 78%, respectively. The results revealed that increasing the methylene blue concentration resulted in a decrease in degradation efficiency. However, the efficiency was still higher even with 160 mg L^−1^ concentration of methylene blue.

**Fig. 10:**
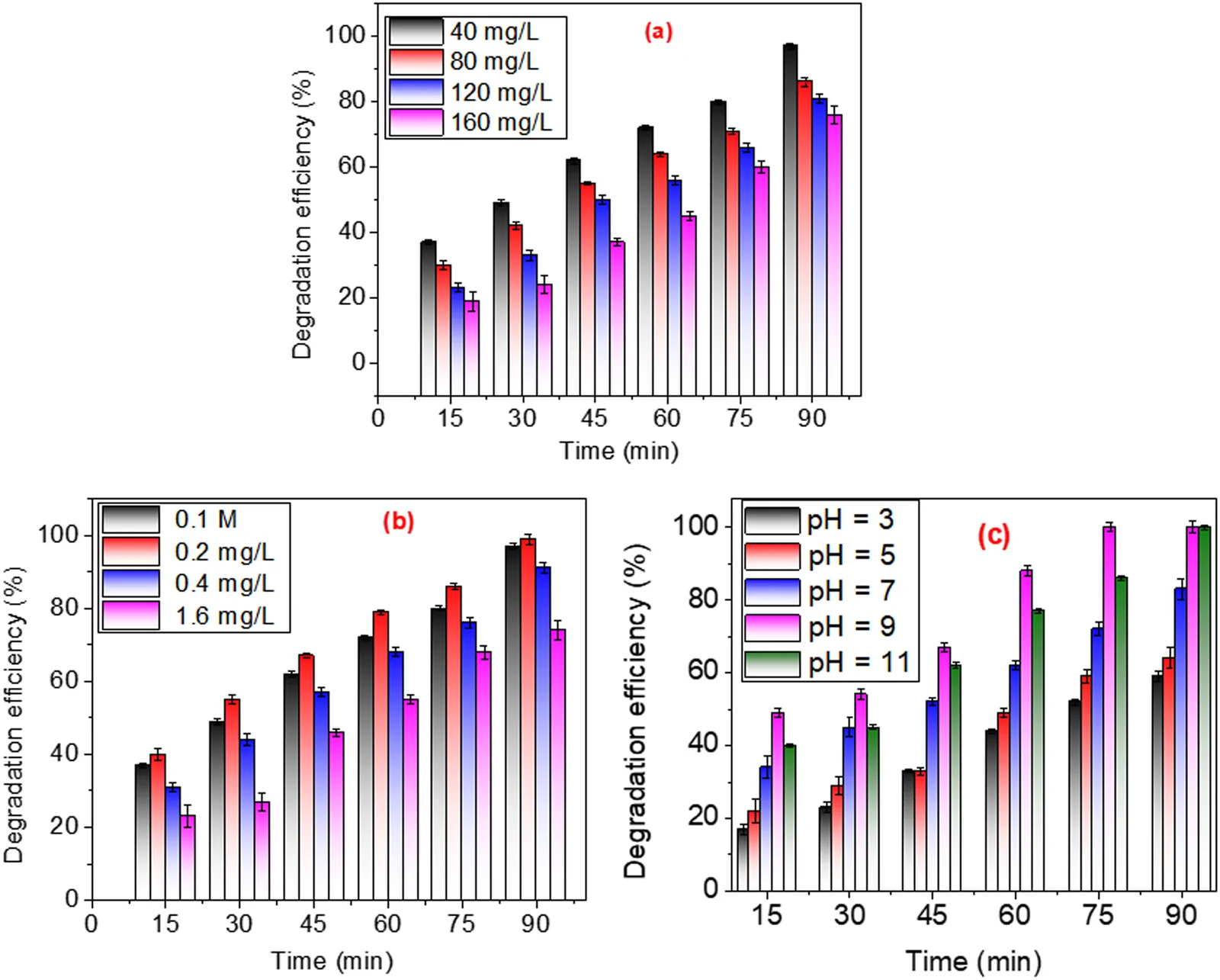
Degradation of methylene blue at different initial concentrations (a), with various PMS concentrations (b) and at various pH values (c).

### 5.2 Effect of the PMS concentration

The effect of the PMS concentration on the degradation efficiency of methylene blue (40 mg L^−1^) was studied using NiFe_2_O_4_/g-C_3_N_4_ under sunlight irradiation. The PMS (1 mL) concentrations of 0.2 M, 0.4 M and 1.6 M were used, and the results were compared with those obtained from 0.1 M under similar conditions. The methylene blue degradation results are shown in Fig. 10b. The methylene blue degradation efficiencies obtained using 0.1 M, 0.2 M, 0.4 M and 1.6 M were 97%, 99%, 91% and 74%, respectively. The results suggest that an increase in the concentration of PMS from 0.1 to 0.2 M demonstrated an increase in methylene blue degradation efficiency from 97% to 99%, respectively. However, a further increase in the PMS concentration to 0.4 M and 1.6 M resulted in a decrease in degradation efficiency. This is caused by the excess PMS quenching the free radicals to form the weaker oxidizing SO_5_^−^ free radicals, as shown in eqn (4) and (5).^44^

4 HSO_5_^−^ + SO_4_˙^−^ → SO_5_^−^ + SO_4_^2−^ + H^+^

5 HSO_5_^−^ + ˙OH → SO_5_^−^ + H_2_O

### 5.3 Effect of pH

The effect of the pH of the solution on the degradation efficiency of methylene blue (40 mg L^−1^) was studied using NiFe_2_O_4_/g-C_3_N_4_ and 0.2 M of PMS under sunlight irradiation. The studies were performed at pH 3, 5, 7, and 11. The plots are shown in Fig. 10c. The degradation efficiency obtained at pH 3, 5, 7, 9 and 11 were 59%, 64%, 83%, 100% and 100%, respectively. The complete degradation efficiency was obtained at pH 9 and 11. Under alkaline solutions, an excess of hydroxide ions (OH^−^) promotes the production of radical species. In addition, the hydroxide anions increase the negetive surface charge on the catalyst, thereby facilitating the adsorption of the positively charged methylene blue dye.^45^

## 6 Reactive oxygen species (ROS) scavenging studies

In order to identify the radicals responsible for the degradation of methylene blue, radical quenching experiments were performed using NiFe_2_O_4_/g-C_3_N_4_ in the presence of PMS and sunlight. Methanol (MeOH) was used to quench ˙OH and SO_4_˙^−^ since MeOH has an affinity for both ˙OH and SO_4_˙^−^. *Tert*-butyl alcohol (TBA) was utilized to quench ˙OH. 4-Benzoquinone (BQ) was utilized for O_2_˙^−^ quenching. The methylene blue degradation results in the absence and presence of radical scavengers are compared in Fig. S7 and Table S3. MeOH can quench both ˙OH and SO_4_˙^−^, while TBA has a high affinity for ˙OH and less affinity for SO_4_˙^−^.^46^ When MeOH, TBA and BQ were used as quenchers, methylene blue degradation efficiencies were 64%, 73% and 88%, respectively. MeOH is a highly hydrophilic scavenger; hence, it does not effectively adsorb onto the surface of the catalyst. Hence, potassium iodide (KI) was utilized to quench SO_4_˙^−^. KI is hydrophobic; thus, it can easily adsorb on the catalyst's surface and capture SO_4_˙^−^. When KI was utilized as a radical scavenger, a methylene blue degradation efficiency of 46% was obtained. From the obtained results, it can be postulated that SO_4_˙^−^ was the dominant degradation species responsible for the degradation of methylene blue.

## 7 Catalyst stability test

The catalyst stability was examined by evaluating the degradation efficiency after 5 cycles. The catalyst was separated from the reaction mixture using a magnet, dried in an oven at 80 °C and re-used in the photocatalytic degradation of methylene blue (40 mg L^−1^) at pH 9 using the NiFe_2_O_4_/g-C_3_N_4_ composite. The plot showing the degradation efficiencies obtained after five cycles is shown in Fig. S4. The degradation efficiency obtained after five cycles was 79%. This suggests that the catalyst exhibited high stability even after five cycles, indicating its suitability for industrial applications.

## 8 Reaction mechanism

The proposed mechanism was predicted following a series of analysis. Firstly, the UV-vis absorption behaviour of NiFe_2_O_4_, g-C_3_N_4_ and NiFe_2_O_4_/g-C_3_N_4_ catalysts were compared, as shown in Fig. S2A. The catalysts displayed absorption below 500 nm. The NiFe_2_O_4_/g-C_3_N_4_ composite displayed enhanced absorption compared to the NiFe_2_O_4_ and g-C_3_N_4_ analogues, with an apparent red shift. This suggests that more visible light can be harvested by the NiFe_2_O_4_/g-C_3_N_4_ composite. The band gap energies (*E*_g_) of the NiFe_2_O_4_, g-C_3_N_4_ and NiFe_2_O_4_/g-C_3_N_4_ catalysts were determined using Tauc plots, as shown in Fig. S2B. The band gap energies of the NiFe_2_O_4_, g-C_3_N_4_ and NiFe_2_O_4_/g-C_3_N_4_ catalysts were 1.77 eV, 2.70 eV, and 1.45 eV, respectively. The NiFe_2_O_4_/g-C_3_N_4_ composite has a lower band gap energy than those of NiFe_2_O_4_ and g-C_3_N_4_. This accounts for its enhanced light-harvesting character compared to pristine NiFe_2_O_4_ and g-C_3_N_4_, as observed in the UV-vis spectrum.

Valence band-X-ray photoelectron spectroscopy (VB-XPS) was utilized to determine the valence bands (VBs) of NiFe_2_O_4_ and g-C_3_N_4_. The VB edges of NiFe_2_O_4_ and g-C_3_N_4_ were obtained to be 2.16 eV and 1.63 eV, respectively. The conduction band edges (*E*_CB_) of NiFe_2_O_4_ and g-C_3_N_4_ were obtained using eqn (6).

6 *E*_CB_ = *E*_VB_ − *E*_g_,

where *E*_VB_ is the valence band potential, *E*_CB_ is the conduction band potential and *E*_g_ is the band gap energy. The *E*_CB_ values of NiFe_2_O_4_ and g-C_3_N_4_ were 0.39 V and −1.07 eV, respectively. Based on the positions of the VBs and CBs of NiFe_2_O_4_ and g-C_3_N_4_, two possible types of heterojunctions can be proposed for the separation of the electron–hole pairs. These heterojunctions operate *via* (i) a type-II charge transfer mechanism and (ii) a Z-scheme heterojunction mechanism.

Under light illumination, as shown in Fig. 11 and eqn (7), the electrons in the VBs of NiFe_2_O_4_ and g-C_3_N_4_ become excited to the CBs of NiFe_2_O_4_ and g-C_3_N_4_, respectively. In the type-II heterojunction, the electrons in the CB of g-C_3_N_4_ are transferred to the CB of NiFe_2_O_4_ since the *E*_CB_ of NiFe_2_O_4_ is more positive than that of g-C_3_N_4_. The holes in the VB of NiFe_2_O_4_ are transferred to the VB of g-C_3_N_4_ since the *E*_VB_ of NiFe_2_O_4_ is more positive than that of g-C_3_N_4_. The reduction reaction occurs in the CB of NiFe_2_O_4_ (to produce superoxide radicals, *E*° O_2_/˙O_2_^−^ = −0.33 eV *vs.* NHE), while the oxidation reaction occurs in the VB of g-C_3_N_4_. However, the *E*° for the production of ˙OH is +1.99 eV against NHE; hence, ˙OH is not anticipated to participate in the degradation of methylene blue in the type-II heterojunction.^47,48^

**Fig. 11:**
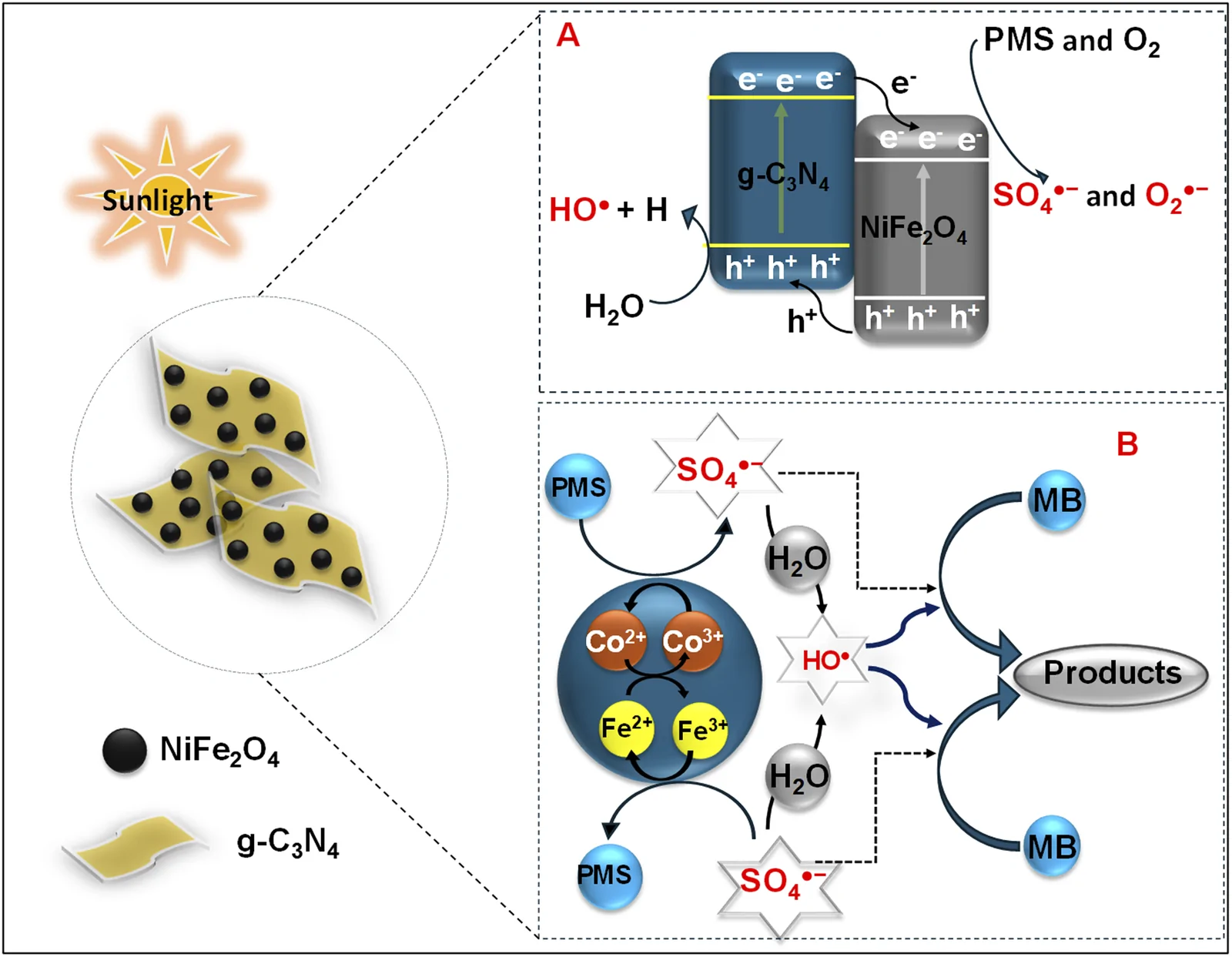
Reaction mechanism for photocatlytic activation of PMS (A), and redox assisted activation of PMS during degradation of methylene blue using the NiFe_2_O_4_/g-C_3_N_4_ heterojunction

However, the ROS quenching studies revealed that ˙OH participated in methylene blue degradation. For this reason, the type-II heterojunction is disregarded, and the Z-scheme heterojunction is proposed, as shown in Fig. 11. The Z-scheme heterojunction suggests that upon irradiation with light and excitation, the electrons in the CB of NiFe_2_O_4_ combine with holes in the VB of g-C_3_N_4_. The electrons accumulated in the CB of g-C_3_N_4_ initiate PMS activation and O_2_ reduction to generate SO_4_˙^−^ and O_2_˙^−^, respectively, as shown in Fig. 11, eqn (8) and (9). The holes in the VB of NiFe_2_O_4_ initiate the oxidation reaction to generate ˙OH, as shown in Fig. 11 and eqn (10). The separation of the photogenerated electron–hole pairs results in enhanced degradation of methylene blue.

7 NiFe_2_O_4_/g-C_3_N_4_ + visible light → NiFe_2_O_4_ (e^−^/h^+^)

8 HSO_5_^−^ + e^−^ → SO_4_˙^−^ + ^−^OH

9 O_2_ + e^−^ → O_2_˙^−^

10 H_2_O + h^+^ → ˙OH + H^+^

Based on ROS scavenging studies, optical analyses and literature reports, the mechanism for PMS activation in the degradation of methylene blue using NiFe_2_O_4_/g-C_3_N_4_ was also proposed.^9,49–51^ Firstly, SO_4_˙^−^ is produced through redox activation of PMS by Ni^2+^ and Fe^2+^, as shown in eqn (11) and(12), respectively. Ni^2+^ and Fe^2+^ are reduced by PMS in the reactions that produce SO_5_˙^−^, as shown in eqn (13) and (14), respectively. At the surface of the NiFe_2_O_4_/g-C_3_N_4_ catalyst, PMS decomposes into SO_5_^2−^ and H^+^, as shown in eqn (15). In the presence of H_2_O, SO_5_^2−^ produces O_2_˙^−^, SO_4_^2−^ and H^+^, as shown in eqn (16). In another process, PMS is activated by light to generate ˙OH and SO_4_˙^−^ species, as shown in eqn (17). Finally, the ROS (˙OH, O_2_˙^−^ and SO_4_˙^−^) participate in the degradation of methylene blue, eqn (18).

11 Fe(ii) + HSO_5_^−^ → Fe(iii) + SO_4_˙^−^ + ^−^OH,

12 Ni(ii) + HSO_5_^−^ → Ni(iii) + SO_4_˙^−^ + ^−^OH,

13 Fe(iii) + HSO_5_^−^ → Fe(ii) + SO_5_˙^−^ + H^+^,

14 Ni(iii) + HSO_5_^−^ → Ni(ii) + SO_5_˙^−^ + H^+^,

15 HSO_5_^−^ → H^+^ + SO_5_^2−^,

16 SO_5_^2−^ + H_2_O → O_2_˙^−^ + SO_4_^2−^ + H^+^,

17 HSO_5_^−^ + light → ˙OH + SO_4_˙^−^,

18 Organic pollutant + O_2_˙^−^ + ˙OH + SO_4_˙^−^ → degradation products.

## 9 Conclusion

A NiFe_2_O_4_/g-C_3_N_4_ Z-scheme heterojunction was successfully synthesized and used for the first time for the activation of PMS to degrade methylene blue in wastewater. The composite showed high photocatalytic activity with a degradation efficiency of 97% after 90 min. SO_4_˙^−^ was the dominant radical species responsible for the degradation of methylene blue. The results showed that NiFe_2_O_4_/g-C_3_N_4_ is a promising catalyst for the removal of organic pollutants from wastewater. We strongly believe that the obtained results may lead to new avenues for designing highly efficient PMS-based photocatalytic technologies for the removal of organic pollutants in wastewater.

## Author contributions

Siphumelele Thandokwazi Mkhondwane: writing – original draft, investigation, conceptualization, methodology, data curation. Yogendra Kumar: methodology, validation. Vinod: methodology, visualisation. Viswanadha Srirama Rajasekhar Pullabhotla: supervision, resources, funding acquisition.

## Conflicts of interest

The authors declare no conflicts of interest.

## Supplementary Material

RA-OLF-D6RA06402G-s001

## Data Availability

Data will be available on request. Supplementary information (SI): plots for Williamson–Hall, UV-vis from adsorption studies, band gaps and valence band edge. See DOI: https://doi.org/10.1039/d6ra06402g.
