# Supplementary material for: Sunlight-assisted activation of peroxymonosulfate using NiFe2O4 supported on a 2D g-C3N4 nanosheet heterojunction for the degradation of methylene blue in wastewater
Source: RSC Adv. 2026 Oct 5;16(46):60143–59. doi: 10.1039/d6ra06402g (PMC13636767; doi:10.1039/d6ra06402g)
Supplement: Supplementary file 1 [file RA-016-D6RA06402G-s001.pdf]

Supplementary Information (SI) for RSC Advances.
This journal is © The Royal Society of Chemistry

Supporting information

For

Sunlight assisted activation of peroxymonosulfate using $NiFe_2O_4$ supported on 2D g-C_3N_4 nanosheets heterojunction for degradation of Methylene blue in wastewater

Siphumelele Thandokwazi Mkhondwane[a*], Yogendra Kumar[b], Vinod[c], and Viswanadha Srirama Rajasekhar Pullabhotla[a]

[a]Department of chemistry, University of Zululand, Private bag X1001, Kwa-Dlangezwa 3886, South Africa.

[b]Department of Chemistry, GLA University, Mathura, Uttar Pradesh, 281406, India.

[c]Department of Chemistry, University of Lucknow, Lucknow-226007, Uttar Pradesh, India.

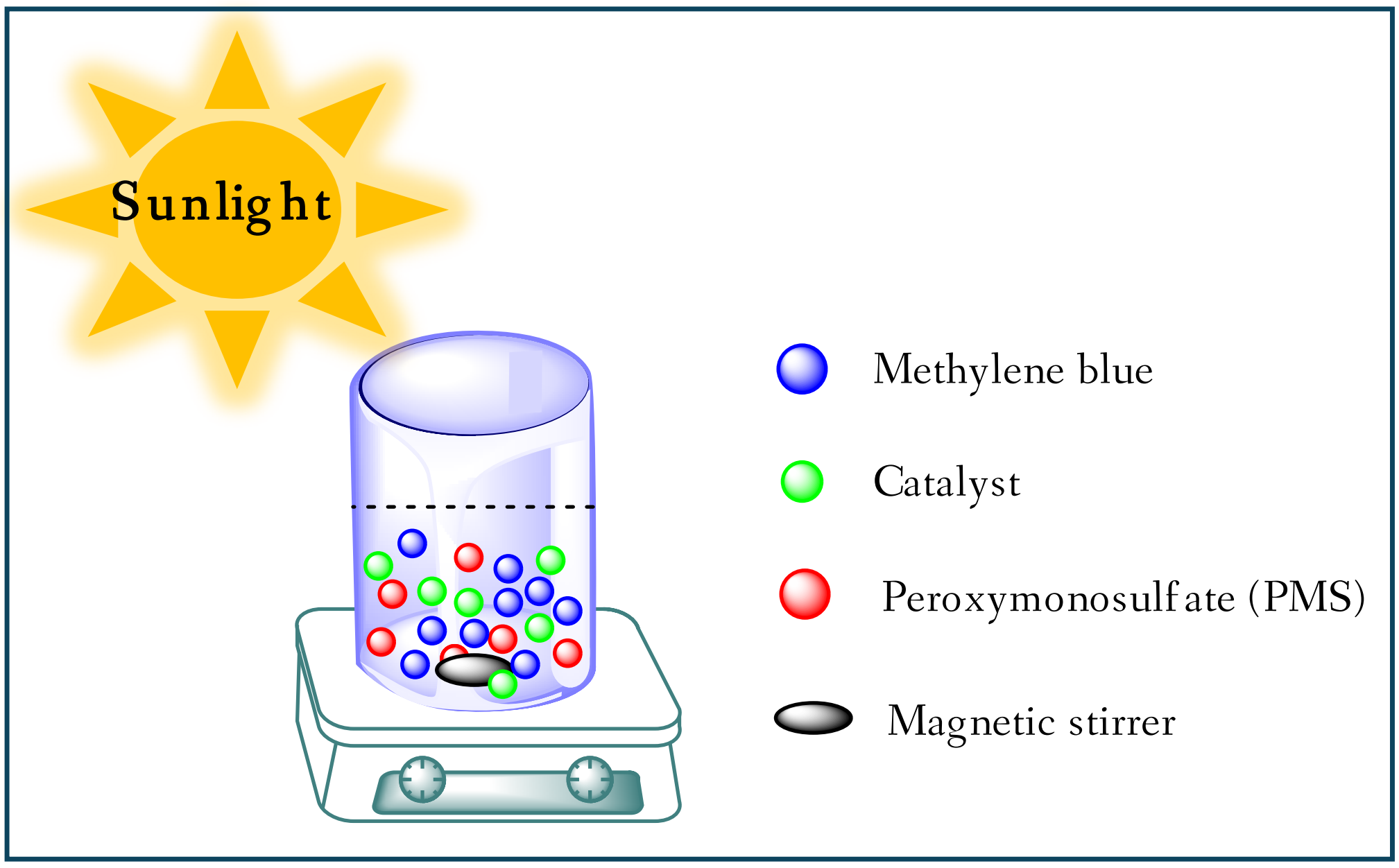

Fig. S1. The reaction setup for methylene blue degradation using $NiFe_2O_4$/g-C_3N_4 catalyst in the presence of PMS.

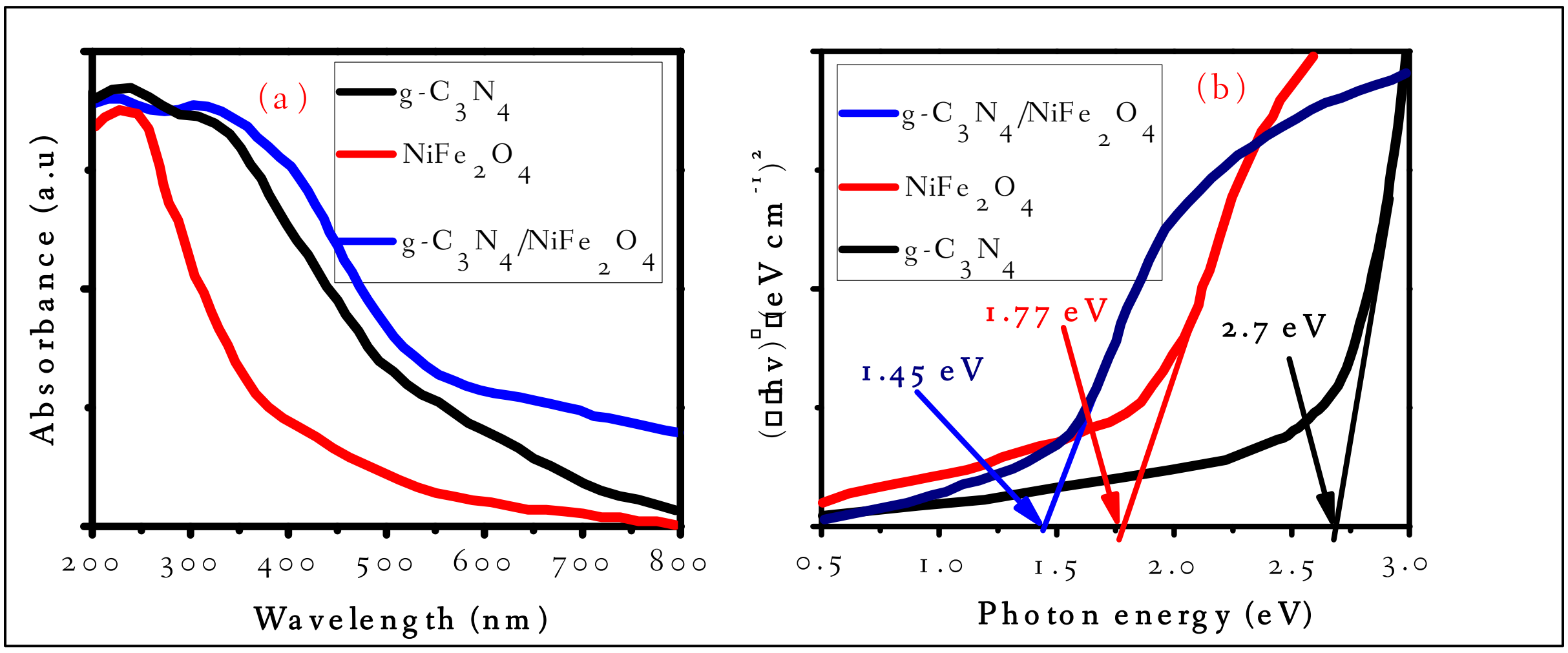

Fig. S1. UV-Vis absorption properties **(a)** and Tauc plots **(b)** of $NiFe_2O_4$, g-C_3N_4 and $NiFe_2O_4$/g-C_3N_4 catalysts.

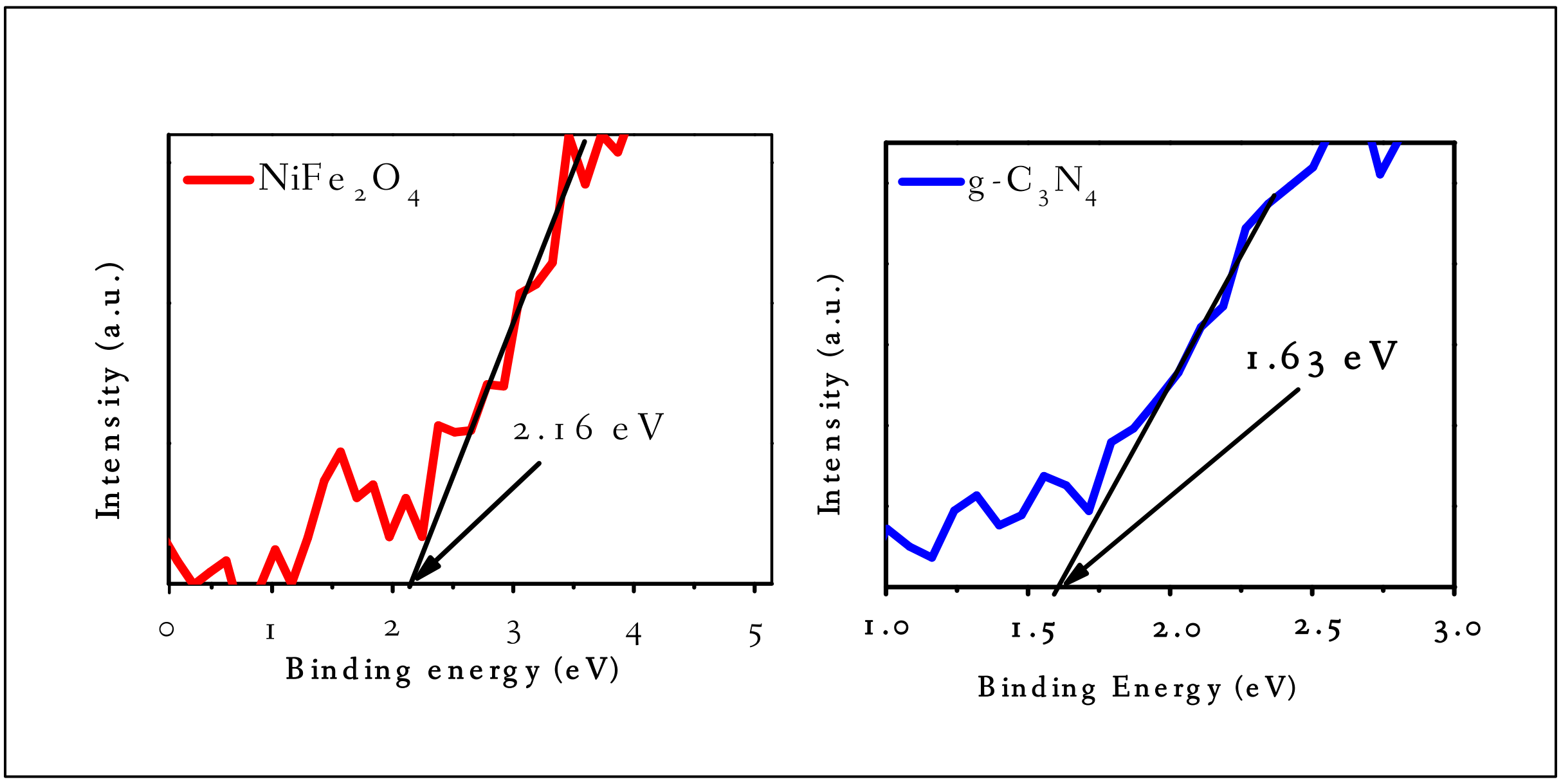

Fig. S3. XPS valence band edge of $NiFe_2O_4$ and g-C_3N_4 catalysts.
